# Cultural competence of undergraduate student nurses: a multicenter study ^*^

**DOI:** 10.1590/1518-8345.7070.4230

**Published:** 2024-07-05

**Authors:** Lucía Sagarra-Romero, Enrique Ramón-Arbués, Isabel Huércanos-Esparza, Indrani Kalkan, Nuran Kömürcü, Valérie Vanceulebroeck, Shana Dehaes, Margarida Coelho, Antonio Casa-Nova, Isabel Antón-Solanas

**Affiliations:** ^1^ San Jorge University, Faculty of Health Sciences, Villanueva de Gállego, Aragón, Spain.; ^2^ İstanbul Medipol Üniversitesi, Kavacık South Campus, Istanbul, Türkiye.; ^3^ Istanbul Aydin University, Nursing Küçükçekmece, Istanbul, Türkiye.; ^4^ AP University, Health and Science, Antwerp, Belgium.; ^5^ Polytechnic Institute of Portalegre, Polytechnic Campus, Portalegre, Portugal.; ^6^ Polytechnic Institute of Portalegre, Health and Sciences Campus, Portalegre, Portugal.; ^7^ University of Zaragoza, Faculty of Healh Sciences, Zaragoza, Aragón, Spain.

**Keywords:** Transcultural Nursing, Cultural Competency, Culturally Competent Care, Nursing Education, Nursing Students, Cross-Sectional Studies

## Abstract

**Objective:**

to evaluate the level of cultural competence of an undergraduate nursing students’ population from four European higher education institutions.

**Method:**

a total of 168 nursing students from four different countries were included in our study. The study methodology involved a cross-sectional assessment of cultural competence among undergraduate nursing students from four European universities. Data collection included sociodemographic variables, as well as the following validated tools: the Intercultural Sensitivity Scale, the Cultural Competence Assessment Tool (student version) and the Cultural Awareness Scale.

**Results:**

our results indicated that students demonstrated a high level of intercultural sensitivity but a moderate level of cultural competence and cultural awareness. Variations existed among students from different countries, suggesting potential differences in educational approaches. Despite expectations that higher-level students would exhibit greater cultural competence, no significant differences were found by year of study, indicating a lack of effective integration of cultural competence into nursing curricula.

**Conclusion:**

nurse educators should consider the students’ cultural competence before designing related study programmes. Training programmes related to cultural competence should include elements which have been associated with enhanced cultural competence, including language skills, cultural encounter, and opportunities for internationalisation.

## Highlights

**(1)** Culturally competent nurses can improve patient outcomes and reduce health inequity.

**(2)** Undergraduate programs should train nurses to become culturally competent nurses.

**(3)** Language skills and cultural encounter are related with greater cultural competence.

## Introduction

 Cultural factors can critically influence the nurse-patient relationship. While it is important that nurses be clinically and technically competent, cultural competence (CC) allows healthcare professionals to engage with culturally diverse patients in a way that respects their cultural identity, thus permitting safe practice and high-quality care. It requires nurses to develop an awareness of their own cultural reality, as well as the attitudes and behaviors that they bring to nursing care; to develop a profound respect and acceptance of the patient’s differences; and to prevent one’s personal beliefs from exerting an undue influence on those whose worldview is non-identical from one’s own ^(^ ^1^ ^)^ . 

 Caring for patients who are culturally diverse is part of most European nurses’ daily routine. Nursing professionals are expected to provide appropriate care to all patients, families and communities, ensuring that their rights are respected regardless of sociocultural identifiers such as race, ethnicity, gender, age and nationality, among others ^(^ ^2^ ^-^ ^3^ ^)^ . CC has been recognized as a bridge that enables nurses to connect to other cultures and understand the skills that are necessary to provide safe patient and family-centered cultural care ^(^ ^1^ ^)^ . 

 In order to ensure health equity for all, nurses must avoid ethnocentric attitudes and cultural bias, become sensitive to every patient’s needs, pay attention to verbal and non-verbal communication, build rapport, engage in cultural negotiation and make efforts to promote patient empowerment, thus creating a sense of safety within the healthcare environment ^(^ ^4^ ^)^ . However, caring for a culturally diverse patient population can be challenging and requires appropriate preparation and training ^(^ ^5^ ^)^ . 

 It is imperative that CC is integrated into nursing education in order to help students develop a culturally mindful and safe practice ^(^ ^6^ ^-^ ^7^ ^)^ . In fact, previous educational interventions seeking to integrate CC in the nursing curriculum have been effective to improve CC in nursing students ^(^ ^8^ ^-^ ^9^ ^)^ . In a systematic review ^(^ ^10^ ^)^ , authors evidenced that CC training had a positive impact on CC of healthcare professionals and was associated with increased patient satisfaction. Thus, it is important to design and implement creative, evidence-based educational activities that promote CC learning outcomes as part of undergraduate nursing programs ^(^ ^11^ ^-^ ^12^ ^)^ . 

 In the European context, higher education institutions (HEIs) are becoming increasingly aware of the need to respond to diverse patient populations. Specifically, it has been established that nursing curricula must provide a foundation for the development of CC that allows the acquisition of knowledge, skills and attitudes ^(^ ^13^ ^)^ . However, the integration of CC into the curriculum of European nursing undergraduate programs is still uneven ^(^ ^14^ ^-^ ^15^ ^)^ . A scoping review of nursing curricula in Spain, Belgium, Portugal, and Turkey revealed a fragmented, non-consistent approach to the concept. For example, in Spain, only 4 out of 94 undergraduate nursing study programs delivered in the academic year 2018-2019 integrated CC as part of their curricula ^(^ ^16^ ^)^ . This is in agreement with the generalized opinion that European nursing curricula lack detailed CC content ^(^ ^1^ ^)^ . However, before implementing any new strategies to address this issue, it is important to measure and analyze European undergraduate student nurses’ current level of CC. Hence, the main purpose of this study was to evaluate the level of CC of an undergraduate nursing students’ population from four European HEIs. 

## Method

### Study design and participants

This study is part of a larger EU-funded project entitled “Transcultural Nursing: A European Priority, a Professional Responsibility for Higher Education” (TC-NURSE), which focuses on cultural, linguistic, and religious diversity, and promotes ownership of shared values, equality, non-discrimination and social inclusion through education and training at higher education level.

 A cross-sectional study of CC was performed on a sample of first to fourth year undergraduate nursing students from four European Universities ( *Universidad San Jorge* , Spain; Istanbul Aydin University, Turkey; *Artis Plantin Hogeschool Antwerpen* , Belgium; *Instituto Politécnico de Portalegre* , Portugal). All the universities included in the study implemented student internationalization programs to promote global awareness, cross-cultural understanding, and international collaboration. As a result, all of them were connected to international student mobility and benefited from several internationalization initiatives, including the ERASMUS program. 

A universal sampling technique was used; that is, all undergraduate student nurses registered in one of the participating universities were invited to participate in the study. The study sample included 168 nursing students from four undergraduate nursing programmes.

The following were inclusion criteria for participant selection: a) undergraduate nursing students registered in one of the participating HEIs; b) be able to read written English. We excluded from our sample any participants who could not communicate in the official language of each country or in English, and those who refused to give informed consent.

An introductory email containing the link to a confidential electronic survey was sent to all the student nurses via university email, explaining the objective of the study, ensuring anonymity and confidentiality of the responses and inviting them to participate. A reminder was sent 2 weeks later. The invitation and data collection were carried out by some of the researchers of the study.

### Data collection

Data were collected between October and November 2021 at the four participating universities.

#### Sociodemographic variables

The first, self-constructed questionnaire contained 18 items and was designed to describe the participants’ sociodemographic characteristics and cultural background. We collected information about the student nurses’ age, gender, occupation, race/ethnicity, religion, socioeconomic level, year of study, clinical work experience, previous CC training and experience working with clients from diverse cultural backgrounds, among other characteristics.

#### Intercultural Sensitivity

 The Intercultural Sensitivity Scale (ISS) contains 24 items ^(^ ^17^ ^)^ . This tool was designed to assess intercultural communication competence and integrates both cross-cultural attitude and behavioural skills. It is based on a 5-point Likert scale (5= strongly agree, 4= agree, 3= uncertain, 2= disagree and 1= strongly disagree). It consists of five factors or constructs on which the statements are based: interaction engagement (7 items), respect for cultural differences (6 items), interaction confidence (5 items), interaction enjoyment (3 items) and interaction attentiveness (3 items). The overall score of the ISS ranges between 24 and 120 with higher scores indicating greater intercultural competency. This scale has demonstrated high internal consistency with .86 reliability coefficient. 

#### Cultural Competence

 The Cultural Competence Assessment Tool (CCATool) in its Student version was used to measure CC in our sample ^(^ ^18^ ^)^ . This instrument is based on the model of development of cultural competence ^(^ ^19^ ^)^ . It includes four sections measuring cultural awareness, cultural knowledge, cultural sensitivity and cultural practice, and a final section of sociodemographic data. This final section was removed from the electronic survey as we measured the sociodemographic and cultural characteristics of our participants through a self-constructed questionnaire. Each of the four sections contains 10 statements measured on a 4-point Likert scale which respondents must agree or disagree with (1= completely disagree, 2= disagree, 3= agree, 4= completely agree) *.* In addition, visual analogue scales (VAS) were included to allow the participants to self-rate their cultural awareness, knowledge, sensitivity, and practice. Validity was confirmed using expert panels; Cronbach alpha reliability coefficient was .70 or higher ^(^ ^20^ ^-^ ^22^ ^)^ . 

#### Cultural Awareness

 Developed in 2003 and reviewed in 2014 ^(^ ^23^ ^-^ ^24^ ^)^ , the Cultural Awareness Scale (CAS) is a 36-item scale measured on a 7-point Likert scale, and includes five constructs or categories of cultural awareness, namely general educational experience, cognitive awareness, research issues, behavior/comfort with interactions and patient care/clinical issues. The internal consistence reliability of the CAS has previously been demonstrated in nursing students ^(^ ^9^ ^,^ ^23^ ^)^ . 

### Data analysis

 Descriptive statistics (mean, standard deviation, and percentage) were calculated to describe the characteristics of the sample. Homogeneity and distribution of the results were examined. When a scale showed a normal distribution, independent sample student *t-* test or ANOVA were used in comparisons; non-parametric tests (Mann-Whitney U test or Kruskal-Wallis) were used when a scale did not show a normal distribution. All statistical analyses were performed using the software Statistical Package for the Social Sciences (version 21.0, SPSS Institute Inc., Chicago, IL, USA). The significance level was set at α = 0.05. 

### Ethical considerations

Eligible participants were informed about the project aims and purpose, and participation in the study was voluntary. Informed consent to participate in the study was assumed when participants completed and sent the electronic survey. Permission to use the measuring tools applied in this study was sought and granted by the authors. The present study was approved by the Research Ethics Committee of Universidad of San Jorge (16-2019) and was carried out in accordance with the principles of the Declaration of Helsinki.

## Results

### Participants’ characteristics

 The participants’ sociodemographic and cultural characteristics are shown in Table 1 . A total of 168 undergraduate nursing students from the study sites responded to the electronic survey. Specifically, 63 students participated from Spain (37.5%), 56 from Turkey (33.5%), 32 from Belgium (19%) and 17 from Portugal (10%). Most of the participants were female (82%), single (86.3%) and mean age was 21.6±3.93 years. 41% of our participants were on their first or second year of study, whereas 59% were on their third or final year. regarding relation to their cultural characteristics, the vast majority of our students described themselves as being from a white background (92.3%); only 3.6% of our students described themselves as being black, 3% as being Asian and 1.2% as being Arab. In terms of their socioeconomic status, our results show that most of our participants were from a middle-class background (85.1%) and resided in an urban area (84.5%). Half of our participants did not have previous clinical work experience and most of them had never attended a CC training course (80.45%). In addition, only 14.3% of the nursing students had lived abroad for at least 3 months. Interestingly, approximately sixty percent of the students described themselves as belonging to a culturally diverse family or group of friends and half of our sample said that they had looked after patients from a diverse cultural background during their practice placements (51.8%). 

**Table 1:** - Socio demographic and cultural characteristics (n = 168). Zaragoza, Spain; Portalegre, Portugal; Istanbul, Turkey; Antwerp, Belgium, 2021

**Variable**	**number**	**Percent (%)**
Age. Mean±SD ^*^	21.63±3.93	
<21 years	67	39.9
≥21 years	101	60.1
Gender		
Female	139	82.7
Male	29	17.3
Country of study		
Spain	63	37.5
Turkey	56	33.3
Belgium	32	19.0
Portugal	17	10.1
Year of study		
First year	55	32.7
Second year	14	8.3
Third year	62	36.9
Fourth year	37	22.0
Marital status		
Single	145	86.3
Married/Partner	23	13.7
Occupation		
Study only	131	78.0
Work and study	37	22.0
Race/ethnicity		
Arab race	2	1.2
Caucasian/white	155	92.3
Black	6	3.6
Asian	5	3.0
Religious affiliation		
Islam	62	36.9
Catholicism	62	36.9
Other	19	11.3
Atheism	25	14.9
Residential environment		
Urban	142	84.5
Rural	26	15.5
Social Status		
Lower social class	4	2.4
Middle social class	143	85.1
High social class	21	12.5
Clinical work experience		
No	84	50.0
Yes	84	50.0
Belonging to a culturally diverse family or group of friends		
No	63	37.5
Yes	105	62.5
Prior cultural competence training		
No	135	80.4
Yes	33	19.6
Prior/current voluntary work with patients from diverse cultural backgrounds and organizations		
No	107	63.7
Yes	61	36.3
Experience in caring for patients from diverse cultural backgrounds		
No	81	48.2
Yes	87	51.8
Lived/studied abroad for at least 3 months		
No	144	85.7
Yes	24	14.3

^*^ SD = Standard Deviation

### Intercultural Sensitivity

 The results from the ISS are displayed in Table 2 . The overall mean score was 95.80±10.86. Taking into account the number of items in the ISS subscales, mean scores were slightly higher for the subscales interaction engagement (27.85±3.51), respect for cultural differences (25.27±4.08) and interaction enjoyment (12.47±2.69); comparatively lower mean scores were observed in the subscales interaction confidence (18.75±2.92) and interaction attentiveness (11.61±1.88). 

No significant differences were found in the scores from the subscales interaction confidence, interaction enjoyment and interaction attentiveness between the students by country of study. The Portuguese students showed higher scores across the subscales interaction engagement (29.70±2.84; p=0.029) and respect for cultural differences (p=0.000); the Belgian students also obtained higher scores from the respect for cultural differences subscale compared with the Spanish and Turkish students.

**Table 2:** - Results from Intercultural Sensitivity Scale classified by country of study (n = 168). Zaragoza, Spain; Portalegre, Portugal; Istanbul, Turkey; Antwerp, Belgium, 2021

**Variable**	**Overall**	**Spain**	**Turkey**	**Belgium**	**Portugal**	**p value**
	**Mean±SD** ^*^	**Mean±SD** ^*^	**Mean±SD** ^*^	**Mean±SD** ^*^	**Mean±SD** ^*^	
Overall (24 items)	95.80±10.86	96.49±10.89	94.21±10.62	94.40 ±10.83	101.11±10.64	0.109 ^†^
Interaction engagement (7 items)	27.85±3.51	27.77±3.92	28.08±3.28	26.62±3.02	29.70±2.84	**0.029** ^†^
Respect for cultural differences (6 items)	25.27±4.08	25.12±3.89	24.14±4.38	26.18±3.66	27.88±3.07	**0.000** ^‡^
Interaction confidence (5 items)	18.57±2.92	19.14±2.72	18.05±2.75	18.37±2.99	18.58±3.87	0.274 ^‡^
Interaction enjoyment (3 items)	12.47±2.69	12.74±1.93	12.01±3.30	12.46±2.69	13.00±2.91	0.578 ^‡^
Interaction attentiveness (3 items)	11.61±1.88	11.69±1.90	11.91±1.80	10.75±1.91	11.94±1.63	0.068 ^‡^

^*^ SD = Standard Deviation;

^†^ ANOVA;

^‡^ Kruskal-Wallis

### Cultural Competence

 The results from the CCATool have been presented in Table 3 . The participants scored higher in the cultural awareness (4.06) and cultural practice (4.01) dimensions, and slightly lower in cultural competence (3.81), cultural knowledge (3.69) and cultural sensitivity (3.46). 

The scores obtained by the students from the different European universities were similar. No significant differences were found between the student nurses by country of study, with the exception of the first dimension, cultural awareness. The results indicate that a statistically significant difference exists in the scores obtained by the students on cultural awareness (Spain-Turkey; p=0.023).

**Table 3:** - Cultural Competence Assessment Tool results classified by the students’ country (n = 168). Zaragoza, Spain; Portalegre, Portugal; Istanbul, Turkey; Antwerp, Belgium, 2021

**Variable**	**Overall**	**Spain**	**Turkey**	**Belgium**	**Portugal**	**p value**
	**Mean±SD** ^*^	**Mean±SD** ^*^	**Mean±SD** ^*^	**Mean±SD** ^*^	**Mean±SD** ^*^	
Cultural Competence (40 items)	3.81 (0.34)	3.79 (0.29)	3.84 (0.43)	3.77 (0.29)	3.82 (0.24)	0.768 ^†^
Cultural awareness (10 items)	4.06 (0.47)	4.01 (0.33)	4.14 (0.64)	3.99 (0.42)	4.12 (0.28)	**0.023** ^‡^
Cultural knowledge (10 items)	3.69 (0.44)	3.64 (0.40)	3.69 (0.53)	3.74 (0.39)	3.77 (0.37)	0.687 ^†^
Cultural sensitivity (10 items)	3.46 (0.43)	3.47 (0.35)	3.53 (0.53)	3.38 (0.39)	3.37 (0.40)	0.362 ^†^
Cultural practice (10 items)	4.01 (0.49)	4.05 (0.53)	4.00 (0.52)	3.96 (0.41)	4.01 (0.39)	0.798 ^‡^

^*^ SD = Standard Deviation;

^†^ ANOVA;

^‡^ Kruskal-Wallis

### Cultural Awareness

Table 4 shows the results from the CAS, overall and by categories, by country of study. Mean score for this scale was 188.07±21.45. The Portuguese students presented the highest scores overall and in all the categories, with a statistically significant difference between them and the rest of the student nurses on the CAS score (p=0.006). The Belgian students scored the lowest in general education experience, although no significant differences were found in this category. There was a statistically significant difference between Spanish and Turkish student nurses in cognitive awareness (p=0.005), and between Spanish and Belgian students in research issues (p=0.004). No difference was observed in behaviors/comfort between the four groups of student nurses. Finally, a statistically significant difference was observed in the patient care/clinical issues category (Portugal 31.0±2.03; Spain 28.26±3.15; p=0.009). 

**Table 4:** - Cultural Awareness Scale results by country of study (n = 168). Zaragoza, Spain; Portalegre, Portugal; Istanbul, Turkey; Antwerp, Belgium, 2021

**Variable**	**Overall**	**Spain**	**Turkey**	**Belgium**	**Portugal**	**p value**
	**Mean±SD** ^*^	**Mean±SD** ^*^	**Mean±SD** ^*^	**Mean±SD** ^*^	**Mean±SD** ^*^	
General education experience (14 items)	71.58±11.60	72.04±11.54	70.26±10.57	69.78±10.87	77.58±14.97	0.104 ^†^
Cognitive awareness (7 items)	36.25±6.45	37.66±4.32	33.75±7.45	36.81±7.50	38.17±5.24	**0.005** ^‡^
Research issues (4 items)	20.63±3.03	21.65±1.89	20.14±3.40	19.15±3.65	21.23±2.61	**0.004** ^‡^
Behavior/Comfort with interactions (6 items)	31.31±6.39	30.88±6.11	30.91±7.01	31.71±6.40	33.47±5.20	0.467 ^†^
Patient care/Clinical issues (5 items)	28.29±4.68	28.26±3.15	27.91±5.79	27.59±5.65	31.00±2.03	**0.009** ^‡^
Overall (36 items)	188.07±21.45	190.52±14.96	182.98±25.02	185.06±24.72	201.47±16.66	**0.006** ^‡^

^*^ SD = Standard Deviation;

^†^ ANOVA;

^‡^ Kruskal-Wallis

A bivariate analysis of the relationship between the students’ level of intercultural sensitivity, CC and cultural awareness, and their year of study was carried out. Students were divided into two groups: 1) First and second year students (41%), and 2) Third and fourth year students (59%). No significant differences were found between both groups for the CAS (p=0.133), ISS (p=0.570) and CCATool (p=0.624) (data not on the tables).

## Discussion

 The sociodemographic characteristics of our participants reflected those of the majority of undergraduate student nurses registered at the four participating universities. In relation to the students’ cultural features, we found it interesting that the majority described themselves as belonging to a culturally diverse family or group of friends. This is probably a reflection of society in the four countries of this study, but it may also be due to the students adopting a broader understanding of cultural difference as involving not only race and ethnicity but also other characteristics such as age, religion, sexual orientation and geographical area ^(^ ^24^ ^)^ . 

 As a whole, our students achieved a high level of intercultural sensitivity. Similar results were obtained by other authors in three samples of health professions students in Turkey ^(^ ^25^ ^-^ ^26^ ^)^ and Australia ^(^ ^27^ ^)^ . Specifically, it was observed that both medical and nursing students who interacted with people from different cultures and spoke a second language presented higher levels of intercultural sensitivity ^(^ ^25^ ^)^ . Interestingly, all the students in our sample spoke at least one more language (English), and over half of them belonged to a culturally diverse group of friends and/or family and had experience looking after patients from minority cultures through their clinical placements. 

 The level of cultural competence of our participants as measured by the CCATool was moderate. In a study of 295 graduating students in Finland ^(^ ^21^ ^)^ were obtained similar results, with the difference that our students obtained higher levels of cultural knowledge, whereas theirs displayed better levels of cultural competence. It was interesting to observe that whilst over 80% of these participants had had some form of formal CC training, only 20% of our sample had previously attended a CC course. However, an association was stablished in both cases between frequency of cultural encounters and linguistic skills, and higher levels of cultural competence. 

 Our students achieved a moderate level of cultural awareness. In a recent cross-sectional study ^(^ ^27^ ^)^ involving a sample of nurses and nursing students, they observed high levels of cultural awareness among the sample (mean = 4.42; SD = 0.45). This would support our initial observation that CC is not integrated in the curricula, or at least it is not made explicit to the students when cultural issues are addressed in the classroom. 

 We analysed the students’ scores from the three scales separately by country of study. Overall, the Portuguese and the Belgian students achieved higher scores than the Spanish and the Turkish in all the scales. It is possible that these results truly reflect a difference between the four groups of student nurses. However, the fact that fewer undergraduate student nurses from Belgium and Portugal participated in this study may have resulted in a sample of students with a particular interest or inclination for CC and, therefore, potentially presenting a higher level of cultural sensitivity awareness and competence. The fact that health professions students in general, and nursing students in particular, achieve high levels on intercultural sensitive is not surprising. The items comprised in the ISS refer to attitudes and/or qualities that are expected of a nurse, such as “I am open-minded to people from different cultures” and “I respect the ways people from different cultures behave”. It is of course possible that the mean score obtained by our student nurses in the ISS is a true reflection of their level of intercultural sensitivity. However, it is also possible that their responses are partially influenced by their projected image of what a qualified nurse should be or behave like. After all, qualities such as respect, open-mindedness and tolerance are frequently associated with the nursing profession, and all of those would be required to demonstrate a high level of cultural sensitivity ^(^ ^28^ ^)^ . 

Regardless of the motivation behind the students’ responses, this is a highly important finding and one that should be considered when designing materials and teaching and learning activities relating to CC. Based on our findings, it could be argued that undergraduate nursing students are a fertile ground on which to continue growing the knowledge, skills, and attitudes that are necessary to become a culturally competent professional. Appraising the students’ initial competence level is key to the design of learning outcomes which are achievable yet challenging for a group of students; to the preparation of teaching and learning materials and activities which enable the students to acquire new, and continue developing existing, knowledge, skills, and attitudes; and to the fair and adequate evaluation of student learning. Thus, we recommend that university lecturers and clinical tutors take the students’ initial level of intercultural sensitivity into account when planning their teaching sessions.

 Factors such as language competence and cultural encounter are frequently associated with higher levels of cultural competence ^(^ ^29^ ^-^ ^30^ ^)^ . All our students were able to speak English and at least half of them were able to interact with people from diverse cultural backgrounds either during clinical placements or as part of a diverse group of friends or family. These characteristics may have resulted in an overestimation of the intercultural sensitivity, CC, and cultural awareness of our participants. Having said this, it seems clear that promoting our students’ language skills and allowing them to experience cultural encounter, both as part of their study programmes and as complementary activities such as summer schools and elective courses with a focus on internationalisation, could be useful to increase their level of CC. Therefore, we argue opportunities should be provided for nursing students to interact with people from diverse cultural backgrounds, develop language skills, and provide possibilities for internationalization ^(^ ^31^ ^)^ . 

 It was highly interesting to observe that no significant differences existed in the results from the ISS; the CCATool and the CAS by the students’ year of study. We expected our third and final year students to achieve a higher level of CC than that of the first and second years. This was based on the assumption that students on their third and fourth year of study are exposed to a larger and wider range of learning opportunities, including international mobility programmes, internationalization at home, clinical placements, etc. This finding supports our view that CC is not sufficiently integrated into European undergraduate nursing programmes; it would also suggest that current compulsory and elective learning opportunities are either not effective or are not taken advantage of by the students. Previous studies have demonstrated that the integration of CC modules and training courses in the curriculum is effective in promoting CC among undergraduate student nurses ^(^ ^32^ ^-^ ^33^ ^)^ . We suggest that such activities are designed carefully, based on the students’ initial level of CC, and include elements which have been strongly associated with higher CC, including language skills, cultural encounter and opportunities for internationalisation. 

There was a statistically significant difference between Spanish and Turkish student nurses in cognitive awareness. There are several factors that may contribute to the variation in levels of cognitive awareness of cultural competence among student nurses, including students’ personal background and experiences, educational preparation, clinical placements, the effectiveness of teaching methods and instructional materials used to impart CC concepts, the attitudes, beliefs, and teaching practices of nursing faculty, the students’ ability and capacity for self-reflection and critical thinking and, last but not least, exposure to CC training.

Overall, the interplay of personal factors, educational experiences, faculty influence, clinical exposure, and self-directed learning contributes to the variation in levels of cognitive awareness of cultural competence among student nurses. Efforts to enhance cultural competence education and create supportive learning environments can help bridge these disparities and ensure that all students develop the necessary awareness and skills to provide culturally sensitive care.

The core findings of this study showed that nursing students from different European Universities have a high grade of intercultural sensitivity, suggesting that nursing school training programs are attempting to promote some areas of cultural competence, such as to understand, appreciate and respect people from other cultures. However, successful integration of CC into nursing curricula requires a comprehensive approach that may encompass various strategies and considerations, including: 1) Clearly defining specific learning objectives related to cultural competence within the nursing curriculum; 2) Infusing CC content throughout the nursing curriculum rather than confining it to a single course or module; 3) Providing opportunities for students to engage in real-world experiences that expose them to diverse cultures and communities, for example as part of clinical placements in culturally diverse settings; 4) Offering cultural immersion experiences, such as international clinical rotations or community-based projects in multicultural neighborhoods; 5) Collaborating with other healthcare disciplines to incorporate interprofessional education focused on cultural competence; 6) Providing faculty with training and resources to effectively teach cultural competence concepts and facilitate meaningful discussions on cultural diversity in the classroom; 7) Integrating assessment tools and methods to evaluate students’ CC throughout the nursing program; 8) Establishing partnerships with community organizations, cultural centers, and healthcare agencies serving diverse populations, and collaborating with stakeholders who can provide valuable opportunities for students to interact with and learn from individuals from different cultural backgrounds; 9) Providing students with access to resources and support services, such as cultural competence workshops, online training modules, and cultural sensitivity training materials, and 10) Regularly assessing the effectiveness of CC initiatives within the nursing curriculum and solicit feedback from students, faculty, and community partners.

Overall, this research can contribute to the advancement of scientific knowledge by enhancing our understanding of cultural competence development, educational interventions, cross-cultural variations, barriers and facilitators, and assessment methods within nursing education and practice.

 There are some limitations in this study that should be noted. First, the length of the electronic survey was a concern (163 items); some students might have found it onerous to complete, resulting in some degree of response bias. However, we were interested in measuring the students’ intercultural sensitivity as well as their perceived level of cultural competence; the CAS tool allowed us to also evaluate the students’ perception or awareness of cultural competence education and training received at their respective universities. Second, considering the total universe of student nurses registered in the study sites, our sample size was limited. This is particularly true in the case of *Artis Plantin Hogeschool Antwerpen* and *Instituto Politécnico de Portalegre* and may explain the Belgian and Portuguese students’ higher scores in some of the questionnaires to some extent. This may have been prevented by adapting and translating the tools into the four languages of the study, but this option was discarded due to time and budget constraints. Finally, the fact that all our students were able to speak English, and speaking a second language has been associated with higher levels of CC, makes it difficult to generalize our results to the whole population of undergraduate student nurses in the four countries of the study. 

## Conclusion

Undergraduate nursing students from four European Universities achieved a high level of intercultural sensitivity and a moderate level of CC and cultural awareness. Thus, it is highly necessary that nursing educators take into consideration the students’ level of CC prior to designing and implementing any related study programmes.

Nursing students are not a white canvas but a fertile ground on which to continue growing the knowledge, skills and attitudes needed to become culturally competent professionals. Accordingly, future teaching and learning programmes and activities related to CC should be carefully designed and include elements which have been associated with enhanced CC, including language skills, cultural encounter, and opportunities for internationalization.
