# Supplementary material for: Cultural competence of undergraduate student nurses: a multicenter study
Source: Rev Lat Am Enfermagem. 2024 Jul 5;32:e4230. doi: 10.1590/1518-8345.7070.4230 (PMC11251689; doi:10.1590/1518-8345.7070.4230)
Supplement: Translation

# Translation: Competencia cultural de estudiantes de pregrado en enfermería: estudio multicéntrico ^*^

**DOI:** 10.1590/1518-8345.7070.4229

**Authors:** Lucía Sagarra-Romero, Enrique Ramón-Arbués, Isabel Huércanos-Esparza, Indrani Kalkan, Nuran Kömürcü, Valérie Vanceulebroeck, Shana Dehaes, Margarida Coelho, Antonio Casa-Nova, Isabel Antón-Solanas

**Affiliations:** ^1^ San Jorge University, Faculty of Health Sciences, Villanueva de Gállego, Aragón, España.; ^2^ İstanbul Medipol Üniversitesi, Kavacık South Campus, Istanbul, Turquía.; ^3^ Istanbul Aydin University, Nursing Küçükçekmece, Istanbul, Turquía.; ^4^ AP University, Health and Science, Antwerp, Bélgica.; ^5^ Polytechnic Institute of Portalegre, Polytechnic Campus, Portalegre, Portugal.; ^6^ Polytechnic Institute of Portalegre, Health and Sciences Campus, Portalegre, Portugal.; ^7^ University of Zaragoza, Faculty of Healh Sciences, Zaragoza, Aragón, España.

**Keywords:** Enfermería Transcultural, Competencia Cultural, Asistencia Sanitaria Culturalmente Competente, Educación en Enfermería, Estudiantes de Enfermería, Estudios Transversales

## Abstract

**Objetivo:**

evaluar el nivel de competencia cultural de una población de estudiantes de pregrado en enfermería de cuatro instituciones europeas de educación superior.

**Método:**

en nuestro estudio se incluyeron en total 168 estudiantes de enfermería de cuatro países diferentes. La metodología de estudio implicó una evaluación transversal de la competencia cultural de los estudiantes de pregrado en enfermería de cuatro universidades europeas. La recolección de datos incluyó variables sociodemográficas y las siguientes herramientas validadas: la Escala de Sensibilidad Intercultural, la Herramienta de Evaluación de la Competencia Cultural (versión para estudiantes) y la Escala de Conciencia Cultural.

**Resultados:**

los estudiantes demostraron un alto nivel de sensibilidad intercultural pero un nivel moderado de competencia y conciencia cultural. Hubo variaciones entre los estudiantes de diferentes países, lo que sugiere posibles diferencias en los enfoques educativos. A pesar de que se esperaba que los estudiantes de educación superior tuvieran mayor competencia cultural, no se encontraron diferencias significativas por año de estudio, lo que indica que la competencia cultural no se incluye de forma efectiva en los planes de estudio de las carreras de pregrado en enfermería.

**Conclusión:**

los educadores de enfermería deben considerar la competencia cultural de los estudiantes antes de diseñar programas de estudio relacionados con la misma. Los programas de formación relacionados con la competencia cultural deben incluir elementos que se hayan asociado con una mayor competencia cultural, eso incluye habilidades lingüísticas, encuentros culturales y oportunidades de internacionalización.

## Destacados

**(1)** Los enfermeros culturalmente competentes pueden mejorar los resultados de los pacientes y reducir la desigualdad en salud.

**(2)** Los programas de las carreras de grado deben formar enfermeros para que se conviertan en enfermeros culturalmente competentes.

**(3)** Las habilidades lingüísticas y el encuentro cultural se relacionan con una mayor competencia cultural.

## Introducción

 Los factores culturales pueden influir de forma decisiva en la relación enfermero-paciente. Si bien es importante que los enfermeros sean clínica y técnicamente competentes, la competencia cultural (CC) les permite a los profesionales de la salud interactuar con pacientes culturalmente diferentes de una manera que respete su identidad cultural y les permita realizar una práctica segura y brindar atención de alta calidad. Para desarrollar la competencia cultural, es necesario que los enfermeros tomen conciencia de su propia realidad cultural, así como de las actitudes y comportamientos que tienen cuando brindan los cuidados de enfermería; desarrollen un profundo respeto y aceptación de las diferencias del paciente; y eviten que tus creencias personales ejerzan una influencia indebida sobre aquellos cuya visión del mundo no es idéntica a la tuya ^(^ ^1^ ^)^ . 

 Atender a pacientes con diversidad cultural es parte de la rutina diaria de la mayoría de los enfermeros europeos. Se espera que los profesionales de enfermería les brinden una atención adecuada a todos los pacientes, las familias y comunidades, y garanticen que sus derechos sean respetados, independientemente de identificadores socioculturales como raza, etnia, género, edad y nacionalidad, entre otros ^(^ ^2^ ^-^ ^3^ ^)^ . La CC fue reconocida como un puente que les permite a los enfermeros conectarse con otras culturas y comprender las habilidades necesarias para brindar una atención cultural segura, centrada en el paciente y la familia ^(^ ^1^ ^)^ . 

 Para garantizar la equidad en salud para todos, los enfermeros deben evitar actitudes etnocéntricas y prejuicios culturales, ser sensibles a las necesidades de cada paciente, prestar atención a la comunicación verbal y no verbal, establecer una buena relación, participar en negociaciones culturales y esforzarse por promover el empoderamiento del paciente, creando así una sensación de seguridad en el ambiente de salud ^(^ ^4^ ^)^ . Sin embargo, brindar atención a una población de pacientes con diversidad cultural puede ser un desafío y requiere preparación y capacitación adecuadas ^(^ ^5^ ^)^ . 

 Es imperativo incorporar la CC a la educación de enfermería para ayudar a los estudiantes a desarrollar una práctica segura y culturalmente consciente ^(^ ^6^ ^-^ ^7^ ^)^ . De hecho, las intervenciones educativas previas que buscaron incorporar la CC en el currículo de enfermería fueron efectivas para mejorar la CC de los estudiantes de enfermería ^(^ ^8^ ^-^ ^9^ ^)^ . En una revisión sistemática ^(^ ^10^ ^)^ , los autores demostraron que la formación en CC tuvo un impacto positivo en la CC de los profesionales de la salud y se asoció con una mayor satisfacción de los pacientes. Por lo tanto, es importante diseñar e implementar actividades educativas creativas, basadas en la evidencia, que promuevan resultados en el aprendizaje de la CC como parte de los programas de las carreras de pregrado en enfermería ^(^ ^11^ ^-^ ^12^ ^)^ . 

 En el contexto europeo, las instituciones de educación superior (IES) son cada vez más conscientes de que es necesario brindar atención a diferentes poblaciones de pacientes. Específicamente, se estableció que los currículos de enfermería deben brindar una base para desarrollar una CC que permita la adquisición de conocimientos, habilidades y actitudes ^(^ ^13^ ^)^ . Sin embargo, en Europa la CC no se incluye de la misma manera en los planes de estudios de las carreras de pregrado en enfermería ^(^ ^14^ ^-^ ^15^ ^)^ . Una revisión de alcance de los planes de estudio de las carreras de grado en enfermería en España, Bélgica, Portugal y Turquía reveló que el enfoque que adoptan es fragmentado y no es consistente con el concepto. Por ejemplo, en España, solo 4 de los 94 programas de las carreras de pregrado en enfermería impartidos durante 2018-2019 incluyeron la CC en sus planes de estudio ^(^ ^16^ ^)^ . Esto concuerda con la opinión generalizada de que los planes de estudios de enfermería europeos no cuentan con un contenido detallado de la CC ^(^ ^1^ ^)^ . Sin embargo, antes de implementar nuevas estrategias para abordar este problema, es importante medir y analizar el nivel actual de CC de los estudiantes europeos de enfermería. Por lo tanto, el objetivo principal de este estudio fue evaluar el nivel de CC de una población de estudiantes de grado en enfermería de cuatro IES europeas. 

## Método

### Diseño del estudio y participantes

 Este estudio es parte de un proyecto más amplio financiado por la UE titulado “Enfermería transcultural: una prioridad europea, una responsabilidad profesional para la educación superior” (“ *Transcultural Nursing: A European Priority, a Professional Responsibility for Higher Education* ”, TC-NURSE), que se centra en la diversidad cultural, lingüística y religiosa y promueve la apropiación de valores compartidos, la igualdad, la no discriminación y la inclusión social a través de la educación y la formación en el nivel de educación superior. 

 Se llevó a cabo un estudio transversal de la CC en una muestra de estudiantes de enfermería de primero a cuarto año de la carrera de pregrado en enfermería de cuatro universidades europeas (Universidad San Jorge, España; *Istanbul Aydin University* , Turquía; *Artis Plantin Hogeschool Antwerpen* , Bélgica; *Instituto Politécnico de Portalegre* , Portugal). Todas las universidades incluidas en el estudio han implementado programas de internacionalización de estudiantes para promover la conciencia global, el entendimiento intercultural y la colaboración internacional. Como resultado, todas cuentan con el programa de movilidad internacional de estudiantes y se beneficiaron de diversas iniciativas de internacionalización, incluido el programa ERASMUS. 

Se utilizó una técnica de muestreo universal, es decir, se invitó a participar en el estudio a todos los estudiantes de pregrado en enfermería inscriptos en alguna de las universidades participantes. La muestra del estudio incluyó a 168 estudiantes de enfermería de cuatro programas de las carreras de pregrado en enfermería.

Los criterios de inclusión para la selección de los participantes fueron los siguientes: a) estudiantes de pregrado en enfermería inscriptos en una de las IES participantes; b) saber leer inglés. Excluimos de nuestra muestra a los participantes que no podían comunicarse en el idioma oficial de cada país o en inglés y a aquellos que se negaron a dar su consentimiento informado.

 Se les envió a todos los estudiantes de enfermería un correo electrónico introductorio que contenía el *link* a una encuesta electrónica confidencial por medio del correo electrónico de la universidad, les explicaba el propósito del estudio, les garantizaba el anonimato y la confidencialidad de las respuestas y los invitaba a participar. Se les envió un recordatorio dos semanas después. La invitación y recolección de datos fueron llevadas a cabo por algunos de los investigadores del estudio. 

### Recolección de datos

Los datos se recopilaron entre octubre y noviembre de 2021 en las cuatro universidades participantes.

#### Variables sociodemográficas

El primer cuestionario, autoelaborado, contenía 18 ítems y fue diseñado para describir las características sociodemográficas y el bagaje cultural de los participantes. Recopilamos información sobre edad, sexo, ocupación, raza/etnia, religión, nivel socioeconómico, año de estudio, experiencia en trabajo clínico, capacitación previa en CC y experiencia en trabajo con pacientes de diversos orígenes culturales, entre otras características.

#### Sensibilidad Intercultural

 La Escala de Sensibilidad Intercultural ( *Intercultural Sensitivity Scale* , ISS) tiene 24 ítems ^(^ ^17^ ^)^ . Esta herramienta fue diseñada para evaluar la competencia en comunicación intercultural e integra tanto la actitud intercultural como las habilidades comportamientales. Se basa en una escala Likert de 5 puntos (5 = completamente de acuerdo, 4 = de acuerdo, 3 = ni de acuerdo ni en desacuerdo, 2 = en desacuerdo y 1 = completamente en desacuerdo). Consta de cinco factores o constructos en los que se basan las afirmaciones: participación en la interacción (7 ítems), respeto por las diferencias culturales (6 ítems), confianza en la interacción (5 ítems), placer en la interacción (3 ítems) y atención a la interacción (3 ítems). La puntuación general de la ISS oscila entre 24 y 120, y las puntuaciones más altas indican mayor competencia intercultural. Esta escala demostró una alta consistencia interna con un coeficiente de confiabilidad de 0,86. 

#### Competencia Cultural

 Para medir la CC en nuestra muestra se utilizó la Herramienta de Evaluación de la Competencia Cultural ( *Cultural Competence Assessment Tool* , CCATool), versión para estudiantes ^(^ ^18^ ^)^ . Este instrumento se basa en el modelo de desarrollo de competencia cultural ^(^ ^19^ ^)^ . Incluye cuatro secciones que miden la conciencia cultural, el conocimiento cultural, la sensibilidad cultural y la práctica cultural, además de una sección final sobre datos sociodemográficos. La sección final se eliminó de la encuesta electrónica porque medimos las características sociodemográficas y culturales de nuestros participantes mediante un cuestionario autoelaborado. Cada una de las cuatro secciones tiene 10 enunciados que se miden mediante una escala Likert de 4 puntos con las que los encuestados deben estar de acuerdo o en desacuerdo (1= totalmente en desacuerdo, 2= en desacuerdo, 3= de acuerdo, 4= totalmente de acuerdo). Además, se incluyeron escalas visuales analógicas (EVA) para que los participantes pudiesen autoevaluar su conciencia, conocimiento, sensibilidad y práctica cultural. La validez se confirmó mediante paneles de expertos; el coeficiente de confiabilidad alfa de Cronbach fue de 0,70 o más ^(^ ^20^ ^-^ ^22^ ^)^ . 

#### Consciencia Cultural

 La Escala de Conciencia Cultural ( *Cultural Awareness Scale* , CAS), desarrollada en 2003 y revisada en 2014 ^(^ ^23^ ^-^ ^24^ ^)^ , es una escala de 36 ítems, que se basa en una escala Likert de 7 puntos, e incluye cinco constructos o categorías de conciencia cultural, a saber: experiencia educativa general, conciencia cognitiva, preguntas de investigación, comportamiento/comodidad con las interacciones y atención al paciente/cuestiones clínicas. La confiabilidad de la consistencia interna de la CAS fue demostrada previamente en estudiantes de enfermería ^(^ ^9^ ^,^ ^23^ ^)^ . 

### Análisis de datos

 Se calcularon estadísticas descriptivas (media, desviación estándar y porcentaje) para describir las características de la muestra. Se analizó la homogeneidad y distribución de los resultados. Para las escalas con distribución normal, se utilizó la prueba t de Student para muestras independientes o ANOVA para realizar las comparaciones; para las escalas que no presentaban distribución normal, se utilizaron pruebas no paramétricas (prueba U de Mann-Whitney o Kruskal-Wallis). Todos los análisis estadísticos se realizaron con el *software* *Statistical Package for the Social Sciences* (versión 21.0, SPSS Institute Inc., Chicago, IL, EE. UU.). El nivel de significancia se definió como α = 0,05. 

### Consideraciones éticas

A los participantes elegibles se les informaron los objetivos y el propósito del proyecto, y la participación en el estudio fue voluntaria. Se asumió que los participantes dieron su consentimiento informado para participar en el estudio cuando completaron y enviaron la encuesta electrónica. Se les solicitó permiso a los autores para utilizar las herramientas de medición aplicadas en este estudio y lo otorgaron. El presente estudio fue aprobado por el Comité de Ética en Investigación de la Universidad de San Jorge (16-2019) y se realizó de acuerdo con los principios de la Declaración de Helsinki.

## Resultados

### Características de los participantes

 Las características sociodemográficas y culturales de los participantes se muestran en la Tabla 1 . En total, 168 estudiantes de pregrado en enfermería de los lugares en los que se llevó a cabo el estudio respondieron la encuesta electrónica. En definitiva, participaron 63 estudiantes de España (37,5%), 56 de Turquía (33,5%), 32 de Bélgica (19%) y 17 de Portugal (10%). La mayoría de los participantes era del sexo femenino (82%), soltera (86,3%) y con una edad promedio de 21,6±3,93 años. El 41% de nuestros participantes estaban en su primer o segundo año de estudio, mientras que el 59% estaba en su tercer o último año. En cuanto a sus características culturales, la gran mayoría de nuestros estudiantes se describió como de origen blanco (92,3%); sólo el 3,6% de nuestros estudiantes se describió como negro, el 3% como asiático y el 1,2% como árabe. En términos de nivel socioeconómico, nuestros resultados muestran que la mayoría de los participantes era de clase media (85,1%) y vivía en una zona urbana (84,5%). La mitad de nuestros participantes no tenía experiencia clínica previa y la mayoría de ellos nunca había participado en un curso de capacitación en CC (80,45%). Además, sólo el 14,3% de los estudiantes de enfermería había vivido en el extranjero durante al menos tres meses. Es interesante observar que aproximadamente el 60% de los estudiantes se describieron como integrantes de una familia o grupo de amigos con diversidad cultural y la mitad de nuestra muestra afirmó haber atendido a pacientes de diversos orígenes culturales durante la residencia profesional (51,8%). 

**Tabla 1:** - Características sociodemográficas y culturales (n = 168). Zaragoza, España; Portalegre, Portugal; Estambul, Turquía; Amberes, Bélgica, 2021

**Variable**	**Número**	**Porcentaje (%)**
Edad. Media±DE ^*^	21,63±3,93	
<21 años	67	39,9
≥21 años	101	60,1
Género		
Femenino	139	82,7
Masculino	29	17,3
País del estudio		
España	63	37,5
Turquía	56	33,3
Bélgica	32	19,0
Portugal	17	10,1
Año de estudio		
Primer año	55	32,7
Segundo año	14	8,3
Tercer año	62	36,9
Cuarto año	37	22,0
Estado civil		
Soltero	145	86,3
Casado/Pareja	23	13,7
Ocupación		
Solo estudia	131	78,0
Trabaja y estudia	37	22,0
Raza/Etnia		
Raza Árabe	2	1,2
Caucásico/Branco	155	92,3
Negro	6	3,6
Asiático	5	3,0
Religión		
Islam	62	36,9
Catolicismo	62	36,9
Otra	19	11,3
Ateo	25	14,9
Lugar de residencia		
Urbano	142	84,5
Rural	26	15,5
Estatus Social		
Clase social baja	4	2,4
Clase social media	143	85,1
Clase social alta	21	12,5
Experiencia en trabajo clínico		
No	84	50,0
Sí	84	50,0
Integrante de una familia o grupo de amigos con diversidad cultural		
No	63	37,5
Sí	105	62,5
Capacitación previa en competencia cultural		
No	135	80,4
Sí	33	19,6
Trabajo voluntario anterior/actual con pacientes de diversos orígenes culturales y organizaciones		
No	107	63,7
Sí	61	36,3
Experiencia en el cuidado de pacientes de diversos orígenes culturales		
No	81	48,2
Sí	87	51,8
Vivió/estudió en el extranjero durante al menos 3 meses		
No	144	85,7
Sí	24	14,3

^*^ DE = Desviación Estándar

### Sensibilidad Intercultural

 Los resultados de la ISS se presentan en la Tabla 2 . La puntuación media general fue de 95,80 ± 10,86. Teniendo en cuenta el número de ítems de las subescalas de la ISS, las puntuaciones medias fueron ligeramente superiores para las subescalas participación en la interacción (27,85±3,51), respeto por las diferencias culturales (25,27±4,08) y placer en la interacción (12,47±2,69); comparativamente, se observaron puntuaciones medias más bajas en las subescalas confianza en la interacción (18,75±2,92) y atención a la interacción (11,61±1,88). 

No se encontraron diferencias significativas en las puntuaciones de las subescalas confianza en la interacción, placer en la interacción y atención a la interacción entre los estudiantes por país de estudio. Los estudiantes portugueses obtuvieron puntuaciones más altas en las subescalas participación en la interacción (29,70±2,84; p=0,029) y respeto por las diferencias culturales (p=0,000); los estudiantes belgas también obtuvieron puntuaciones más altas en la subescala respeto por las diferencias culturales que los estudiantes españoles y turcos.

**Tabla 2:** - Resultados de la Escala de Sensibilidad Intercultural clasificados por país de estudio (n = 168). Zaragoza, España; Portalegre, Portugal; Estambul, Turquía; Amberes, Bélgica, 2021

**Variable**	**General**	**España**	**Turquía**	**Bélgica**	**Portugal**	**p-valor**
	**Media±DE** ^*^	Media±DE ^*^	Media±DE ^*^	Media±DE ^*^	Media±DE ^*^	
General (24 ítems)	95,80±10,86	96,49±10,89	94,21±10,62	94,40 ±10,83	101,11±10,64	0,109 ^†^
Participación en la interacción (7 ítems)	27,85±3,51	27,77±3,92	28,08±3,28	26,62±3,02	29,70±2,84	**0,029** ^†^
Respeto por las diferencias culturales (6 ítems)	25,27±4,08	25,12±3,89	24,14±4,38	26,18±3,66	27,88±3,07	**0,000** ^‡^
Confianza en la interacción (5 ítems)	18,57±2,92	19,14±2,72	18,05±2,75	18,37±2,99	18,58±3,87	0,274 ^‡^
Placer en la interacción (3 ítems)	12,47±2,69	12,74±1,93	12,01±3,30	12,46±2,69	13,00±2,91	0,578 ^‡^
Atención a la interacción (3 ítems)	11,61±1,88	11,69±1,90	11,91±1,80	10,75±1,91	11,94±1,63	0,068 ^‡^

^*^ DE = Desviación Estándar;

^†^ ANOVA;

^‡^ Kruskal-Wallis

### Competencia Cultural

 Los resultados de la CCATool se presentan en la Tabla 3 . Los participantes obtuvieron las puntuaciones más altas en las dimensiones conciencia cultural (4,06) y práctica cultural (4,01), y ligeramente más bajas en competencia cultural (3,81), conocimiento cultural (3,69) y sensibilidad cultural (3,46). 

Las puntuaciones obtenidas por los estudiantes de diferentes universidades europeas fueron similares. No se encontraron diferencias significativas entre los estudiantes de enfermería según el país de estudio, excepto en la primera dimensión, conciencia cultural. Los resultados indican que existe una diferencia estadísticamente significativa en las puntuaciones obtenidas por los estudiantes en conciencia cultural (España-Turquía; p=0,023).

**Tabla 3:** - Resultados de la Herramienta de Evaluación de la Competencia Cultural clasificados según el país de los estudiantes (n=168). Zaragoza, España; Portalegre, Portugal; Estambul, Turquía; Amberes, Bélgica, 2021

**Variable**	**General**	**España**	**Turquía**	**Bélgica**	**Portugal**	**p valor**
	**Media±DE** ^*^	Media±DE ^*^	Media±DE ^*^	Media±DE ^*^	Media±DE ^*^	
Competencia cultural (40 ítems)	3,81 (0,34)	3,79 (0,29)	3,84 (0,43)	3,77 (0,29)	3,82 (0,24)	0,768 ^†^
Consciencia cultural (10 ítems)	4,06 (0,47)	4,01 (0,33)	4,14 (0,64)	3,99 (0,42)	4,12 (0,28)	**0,023** ^‡^
Conocimiento cultural (10 ítems)	3,69 (0,44)	3,64 (0,40)	3,69 (0,53)	3,74 (0,39)	3,77 (0,37)	0,687 ^†^
Sensibilidad cultural (10 ítems)	3,46 (0,43)	3,47 (0,35)	3,53 (0,53)	3,38 (0,39)	3,37 (0,40)	0,362 ^†^
Práctica cultural (10 ítems)	4,01 (0,49)	4,05 (0,53)	4,00 (0,52)	3,96 (0,41)	4,01 (0,39)	0,798 ^‡^

^*^ DE = Desviación Estándar;

^†^ ANOVA;

^‡^ Kruskal-Wallis

### Conciencia Cultural

 La Tabla 4 muestra los resultados de la CAS, en general y por categorías, por país de estudio. La puntuación media de esta escala fue de 188,07±21,45. Los estudiantes portugueses obtuvieron las puntuaciones más altas en general y en todas las categorías, hubo una diferencia estadísticamente significativa entre ellos y el resto de los estudiantes de enfermería en la puntuación de la CAS (p=0,006). Los estudiantes belgas obtuvieron las puntuaciones más bajas en experiencia educativa general, aunque no hubo diferencias significativas en esta categoría. Se registró una diferencia estadísticamente significativa entre los estudiantes de enfermería españoles y turcos en conciencia cognitiva (p=0,005), y entre los estudiantes españoles y belgas en preguntas de investigación (p=0,004). No se observaron diferencias en comportamiento/comodidad entre los cuatro grupos de estudiantes de enfermería. Por último, se observó una diferencia estadísticamente significativa en la categoría atención al paciente/cuestiones clínicas (Portugal 31,0±2,03; España 28,26±3,15; p=0,009). 

**Tabla 4:** - Resultados de la Escala de Conciencia Cultural por país de estudio (n=168). Zaragoza, España; Portalegre, Portugal; Estambul, Turquía; Amberes, Bélgica, 2021

**Variable**	**General**	**España**	**Turquía**	**Bélgica**	**Portugal**	**p valor**
	**Media±DE** ^*^	Media±DE ^*^	Media±DE ^*^	Media±DE ^*^	Media±DE ^*^	
Experiencia en educación General (14 ítems)	71,58±11,60	72,04±11,54	70,26±10,57	69,78±10,87	77,58±14,97	0,104 ^†^
Consciencia cognitiva (7 ítems)	36,25±6,45	37,66±4,32	33,75±7,45	36,81±7,50	38,17±5,24	**0,005** ^‡^
Preguntas de investigación (4 ítems)	20,63±3,03	21,65±1,89	20,14±3,40	19,15±3,65	21,23±2,61	**0,004** ^‡^
Comportamiento/Comodidad con las interacciones (6 ítems)	31,31±6,39	30,88±6,11	30,91±7,01	31,71±6,40	33,47±5,20	0,467 ^†^
Atención al paciente/cuestiones clínicas (5 ítems)	28,29±4,68	28,26±3,15	27,91±5,79	27,59±5,65	31,00±2,03	**0,009** ^‡^
General (36 ítems)	188,07±21,45	190,52±14,96	182,98±25,02	185,06±24,72	201,47±16,66	**0,006** ^‡^

^*^ DE = Desviación Estándar;

^†^ ANOVA;

^‡^ Kruskal-Wallis

Se realizó un análisis bivariado de la relación entre el nivel de sensibilidad intercultural, la CC y la conciencia cultural de los estudiantes y su año de estudio. Los estudiantes se dividieron en dos grupos: 1) estudiantes de primer y segundo año (41%) y 2) estudiantes de tercer y cuarto año (59%). No se encontraron diferencias significativas entre los dos grupos para la CAS (p=0,133), la ISS (p=0,570) y la CCATool (p=0,624) (datos no presentados en tablas).

## Discusión

 Las características sociodemográficas de nuestros participantes reflejaron las de la mayoría de los estudiantes de pregrado en enfermería inscriptos en las cuatro universidades participantes. En cuanto a las características culturales de los estudiantes, nos pareció interesante que la mayoría se describió como integrante de una familia o grupo de amigos con diversidad cultural. Es probable que eso sea un reflejo de la sociedad en los cuatro países de este estudio, pero también puede deberse a que los estudiantes tienen una comprensión más amplia de la diferencia cultural, que incluye no sólo la raza y el origen étnico, sino también otras características como la edad, la religión, la orientación sexual y el área geográfica ^(^ ^24^ ^)^ . 

 En general, nuestros estudiantes han alcanzado un alto nivel de sensibilidad intercultural. Otros autores obtuvieron resultados similares en tres muestras de estudiantes de profesiones de salud en Turquía ^(^ ^25^ ^-^ ^26^ ^)^ y Australia ^(^ ^27^ ^)^ . Se observó, particularmente, que tanto los estudiantes de medicina como de enfermería que interactuaban con personas de diferentes culturas y hablaban una segunda lengua presentaron mayores niveles de sensibilidad intercultural ^(^ ^25^ ^)^ . Es interesante notar que todos los estudiantes de nuestra muestra hablaban al menos otro idioma (inglés), y más de la mitad de ellos pertenecía a un grupo de amigos y/o familiares con diversidad cultural y tenía experiencia en la atención de pacientes de culturas minoritarias en la residencia profesional. 

 El nivel de competencia cultural de nuestros participantes, medido por la CCATool, era moderado. En un estudio con 295 estudiantes de grado en Finlandia ^(^ ^21^ ^)^ , obtuvimos resultados similares, pero nuestros estudiantes obtuvieron mayores niveles de conocimiento cultural, mientras que los suyos presentaron mayores niveles de competencia cultural. Fue interesante observar que, aunque más del 80% de estos participantes había tenido algún tipo de capacitación formal en CC, sólo el 20% de nuestra muestra había participado alguna vez en un curso de CC. Sin embargo, en ambos casos se estableció una asociación entre la frecuencia de encuentros culturales y habilidades lingüísticas y niveles más altos de competencia cultural. 

 Nuestros estudiantes han alcanzado un nivel moderado de conciencia cultural. En un estudio transversal reciente ^(^ ^27^ ^)^ , cuyos participantes eran enfermeros y estudiantes de enfermería, se observaron altos niveles de conciencia cultural en la muestra (media = 4,42; DE = 0,45). Esto coincidiría con nuestra observación inicial, que la CC no forma parte de los planes de estudio o, al menos, no se les explicita a los alumnos cuando se abordan cuestiones culturales en el aula. 

 Analizamos las puntuaciones de los estudiantes en las tres escalas por separado según el país de estudio. En general, los estudiantes portugueses y belgas obtuvieron puntuaciones más altas que los españoles y turcos en todas las escalas. Es posible que estos resultados en realidad reflejen una diferencia entre los cuatro grupos de estudiantes de enfermería. Sin embargo, el hecho de que participara un número menor de estudiantes de enfermería de Bélgica y Portugal en este estudio puede haber dado como resultado una muestra de estudiantes con un interés o inclinación especial por la CC y, por lo tanto, potencialmente, presenten un mayor nivel de sensibilidad y competencia cultural. No es una sorpresa que los estudiantes de profesiones de la salud en general, y de enfermería en particular, alcancen altos niveles de sensibilidad intercultural. Los ítems presentes en la ISS corresponden a actitudes y/o cualidades que se esperan de un enfermero, como “tengo una mente abierta hacia personas de diferentes culturas” y “respeto el comportamiento de las personas de diferentes culturas”. Es posible, por supuesto, que la puntuación media obtenida por nuestros estudiantes de enfermería en la ISS sea un fiel reflejo de su nivel de sensibilidad intercultural. Sin embargo, también es posible que sus respuestas estén parcialmente influenciadas por la imagen preestablecida de cómo debería ser o comportarse un enfermero calificado. Después de todo, cualidades como el respeto, la mentalidad abierta y la tolerancia a menudo se asocian con la profesión de enfermería, y todas ellas serían necesarias para demostrar un alto nivel de sensibilidad cultural ^(^ ^28^ ^)^ . 

Independientemente de la motivación detrás de las respuestas de los estudiantes, este es un hallazgo muy importante que debe tenerse en cuenta al diseñar materiales y actividades de enseñanza y aprendizaje relacionados con la CC. Según nuestros hallazgos, se puede afirmar que los estudiantes de grado en enfermería son un terreno fértil para continuar desarrollando el conocimiento, las habilidades y las actitudes necesarias para convertirse en un profesional culturalmente competente. Es fundamental evaluar el nivel de competencia inicial de los estudiantes para diseñar resultados de aprendizaje que sean alcanzables pero desafiantes para un grupo de estudiantes; para elaborar materiales y actividades de enseñanza y aprendizaje que les permitan a los estudiantes adquirir nuevos conocimientos, habilidades y actitudes y seguir desarrollando los que tienen; y para evaluar de forma justa y adecuada el aprendizaje de los estudiantes. Por ello, recomendamos que los profesores universitarios y los tutores clínicos tengan en cuenta el nivel inicial de sensibilidad intercultural de los alumnos a la hora de planificar las clases.

 Factores como la competencia lingüística y el encuentro cultural a menudo se asocian con niveles más altos de competencia cultural ^(^ ^29^ ^-^ ^30^ ^)^ . Todos nuestros estudiantes podían hablar inglés y al menos la mitad de ellos pudo interactuar con personas de diversos orígenes culturales durante su residencia profesional o como integrante de un grupo con diversidad cultural de amigos o familiares. Estas características pueden haber llevado a que se sobreestime la sensibilidad intercultural, la CC y la conciencia cultural de nuestros participantes. Por ende, es obvio que potenciar las habilidades lingüísticas de nuestros estudiantes y permitirles vivir encuentros culturales, ya sea como parte de sus programas de estudio o como actividades complementarias, tales como escuelas de verano y cursos optativos centrados en la internacionalización, puede ser útil para incrementar su nivel de CC. Por lo tanto, consideramos que hay brindarles a los estudiantes de enfermería la oportunidad de interactuar con personas de diversos orígenes culturales, desarrollar habilidades lingüísticas y la posibilidad de internacionalización ^(^ ^31^ ^)^ . 

 Fue muy interesante observar que no hubo diferencias significativas en los resultados de la ISS, CCATool y CAS en lo que respecta al año de estudio de los estudiantes. Esperábamos que nuestros alumnos de tercer y cuarto año tuvieran un nivel más alto de CC que nuestros alumnos de primero y segundo año. Esto se basaba en el supuesto de que los estudiantes de tercer y cuarto año están expuestos a una gama cada vez mayor de oportunidades de aprendizaje, eso incluye programas de movilidad internacional, internacionalización en casa, residencia profesional, y otros. Este hallazgo coincide con nuestra opinión sobre que la CC no tiene la suficiente presencia en los programas europeos de las carreras de grado en enfermería; eso también sugiere que las actuales oportunidades de aprendizaje obligatorio y optativo no son efectivas o los estudiantes no las aprovechan. Estudios anteriores demuestran que la inclusión de módulos de CC y de cursos de capacitación en el plan de estudios son eficaces para promover la CC en los estudiantes de pregrado en enfermería ^(^ ^32^ ^-^ ^33^ ^)^ . Sugerimos que estas actividades se diseñen cuidadosamente, en función del nivel inicial de CC de los estudiantes, e incluyan elementos que se asocien significativamente con una mayor CC, eso incluye habilidades lingüísticas, encuentros culturales y oportunidades de internacionalización. 

Hubo una diferencia estadísticamente significativa entre los estudiantes de enfermería españoles y turcos en lo que respecta a la conciencia cognitiva. Hay varios factores que pueden contribuir a la variación de los niveles de conciencia cognitiva de la competencia cultural de los estudiantes de enfermería, entre ellos, los antecedentes y experiencias personales de los estudiantes, la preparación educativa, la residencia profesional, la eficacia de los métodos y materiales institucionales de enseñanza utilizados para transmitir los conceptos de CC, las actitudes, creencias y prácticas de enseñanza de los profesores de enfermería, la habilidad y capacidad de autorreflexión y pensamiento crítico de los estudiantes y, por último, pero no menos importante, la exposición a la capacitación en CC.

En general, la interacción de factores personales, experiencias educativas, influencia de los profesores, residencia profesional y aprendizaje autodirigido contribuye a que varíen los niveles de conciencia cognitiva de la competencia cultural de los estudiantes de enfermería. Los esfuerzos para mejorar la educación sobre competencia cultural y crear entornos de aprendizaje propicios pueden ayudar a eliminar esas disparidades y garantizar que todos los estudiantes desarrollen la conciencia y las habilidades necesarias para brindar una atención culturalmente sensible.

 Los principales resultados de este estudio mostraron que los estudiantes de enfermería de diferentes universidades europeas tienen un alto grado de sensibilidad intercultural, lo que sugiere que los programas de formación de las escuelas de enfermería intentan promover algunas características de la competencia cultural, como comprender, apreciar y respetar a las personas de otras culturas. Sin embargo, la inclusión exitosa de la CC en los planes de estudios de enfermería requiere un abordaje integral que puede incluir varias estrategias y consideraciones, entre las que se encuentran: 1) Definir claramente los objetivos de aprendizaje específicos relacionados con la competencia cultural dentro del plan de estudios de enfermería; 2) Incluir el contenido de CC en todo el plan de estudios de enfermería en lugar de limitarlo a un solo curso o módulo; 3) Brindar oportunidades para que los estudiantes participen en experiencias del mundo real que los expongan a diversas culturas y comunidades, por ejemplo, realizar la residencia profesional en entornos culturalmente diversos; 4) Brindar experiencias de inmersión cultural, como rotaciones clínicas internacionales o proyectos comunitarios en vecindarios multiculturales; 5) Colaborar con otras disciplinas de la salud para incorporar la educación interprofesional centrada en la competencia cultural; 6) Proporcionarles a los profesores capacitación y recursos para enseñar eficazmente los conceptos de competencia cultural y facilitar debates significativos sobre la diversidad cultural en el aula; 7) Integrar herramientas y métodos de evaluación para evaluar la CC de los estudiantes a lo largo del programa de enfermería; 8) Establecer asociaciones con organizaciones comunitarias, centros culturales y agencias de salud que atienden a poblaciones con diversidad cultural y colaboran con las partes interesadas que pueden brindar oportunidades valiosas para que los estudiantes interactúen y aprendan de personas de diferentes orígenes culturales; 9) Proporcionarles a los estudiantes acceso a recursos y servicios de apoyo, como talleres de competencia cultural, módulos de capacitación *online* y materiales de capacitación en sensibilidad cultural; y 10) Evaluar periódicamente la eficacia de las iniciativas de la inclusión de la CC en el plan de estudios de enfermería y solicitar el *feedback* de los estudiantes, los profesores y las organizaciones comunitarias. 

En general, esta investigación puede contribuir al avance del conocimiento científico al mejorar nuestra comprensión sobre el desarrollo de la competencia cultural, las intervenciones educativas, las variaciones interculturales, las barreras y los facilitadores, y los métodos de evaluación en la educación y la práctica de enfermería.

 El presente estudio tiene algunas limitaciones que hay que tener en cuenta. En primer lugar, nos preocupa el tamaño de la encuesta electrónica (163 ítems); es posible que algunos estudiantes consideraran que era mucho trabajo completar la tarea, lo que puede haber generado cierto grado de sesgo de respuesta. Pero, estábamos interesados en medir la sensibilidad intercultural de los estudiantes, al igual que su nivel percibido de competencia cultural; la herramienta CAS nos permitió evaluar también la percepción o la concientización de los estudiantes sobre la educación y formación en competencias culturales que recibieron en sus respectivas universidades. En segundo lugar, considerando el total de estudiantes de enfermería inscriptos en los lugares que se llevó a cabo el estudio, el tamaño de la nuestra fue limitado. Esto se puede ver particularmente en el caso del *Artis Plantin Hogeschool Antwerpen* y el *Instituto Politécnico de Portalegre* y puede explicar, hasta cierto punto, las puntuaciones más altas de los estudiantes belgas y portugueses en algunos de los cuestionarios. Esto podría haberse evitado adaptando y traduciendo las herramientas a los cuatro idiomas del estudio, pero esta opción se descartó debido a limitaciones de tiempo y presupuesto. Por último, el hecho de que todos nuestros estudiantes hablen inglés, y el hecho de que hablar un segundo idioma se haya asociado con niveles más altos de CC, dificulta generalizar nuestros resultados a toda la población de estudiantes de pregrado en enfermería en los cuatro países del estudio. 

## Conclusión

Los estudiantes de enfermería de cuatro universidades europeas lograron un alto nivel de sensibilidad intercultural y un nivel moderado de CC y conciencia cultural. Por lo tanto, es muy necesario que los educadores de enfermería tengan en cuenta el nivel de CC de los estudiantes antes de diseñar e implementar cualquier programa de estudio relacionado con la misma.

Los estudiantes de enfermería no son un lienzo en blanco, sino un terreno fértil en el que pueden seguir desarrollando los conocimientos, las habilidades y las actitudes necesarias para convertirse en profesionales culturalmente competentes. Por ende, los futuros programas y actividades de enseñanza y aprendizaje relacionados con la CC deben diseñarse cuidadosamente e incluir elementos que se hayan asociado con una mayor CC, eso incluye habilidades lingüísticas, encuentros culturales y oportunidades de internacionalización.

# Translation: Competência cultural de estudantes de graduação em enfermagem: um estudo multicêntrico ^*^

**DOI:** 10.1590/1518-8345.7070.4231

**Authors:** Lucía Sagarra-Romero, Enrique Ramón-Arbués, Isabel Huércanos-Esparza, Indrani Kalkan, Nuran Kömürcü, Valérie Vanceulebroeck, Shana Dehaes, Margarida Coelho, Antonio Casa-Nova, Isabel Antón-Solanas

**Affiliations:** ^1^ San Jorge University, Faculty of Health Sciences, Villanueva de Gállego, Aragón, Espanha.; ^2^ İstanbul Medipol Üniversitesi, Kavacık South Campus, Istanbul, Turquia.; ^3^ Istanbul Aydin University, Nursing Küçükçekmece, Istanbul, Turquia.; ^4^ AP University, Health and Science, Antwerp, Bélgica.; ^5^ Polytechnic Institute of Portalegre, Polytechnic Campus, Portalegre, Portugal.; ^6^ Polytechnic Institute of Portalegre, Health and Sciences Campus, Portalegre, Portugal.; ^7^ University of Zaragoza, Faculty of Healh Sciences, Zaragoza, Aragón, Espanha.

**Keywords:** Enfermagem Transcultural, Competência Cultural, Assistência à Saúde Culturalmente Competente, Educação em Enfermagem, Estudantes de Enfermagem, Estudos Transversais

## Abstract

**Objetivo:**

avaliar o nível de competência cultural de uma população de estudantes de graduação em enfermagem de quatro instituições europeias de ensino superior.

**Método:**

um total de 168 estudantes de enfermagem de quatro países diferentes foram incluídos em nosso estudo. A metodologia do estudo envolveu uma avaliação transversal da competência cultural entre estudantes de graduação em enfermagem de quatro universidades europeias. A coleta de dados incluiu variáveis sociodemográficas, bem como as seguintes ferramentas validadas: Escala de Sensibilidade Intercultural, Ferramenta de Avaliação de Competência Cultural (versão do aluno) e Escala de Consciência Cultural.

**Resultados:**

nossos resultados indicaram que os alunos demonstraram um alto nível de sensibilidade intercultural, mas um nível moderado de competência e consciência cultural. Houve variações entre os alunos de diferentes países, o que sugere possíveis diferenças nas abordagens educacionais. Apesar das expectativas de que os alunos de nível superior apresentariam maior competência cultural, não foram encontradas diferenças significativas por ano de estudo, indicando uma falta de integração eficaz da competência cultural nos currículos de enfermagem.

**Conclusão:**

os educadores de enfermagem devem considerar a competência cultural dos alunos antes de elaborar programas de estudo relacionados. Os programas de treinamento relacionados à competência cultural devem incluir elementos que tenham sido associados a uma maior competência cultural, incluindo habilidades linguísticas, encontros culturais e oportunidades de internacionalização.

## Destaques

**(1)** Os enfermeiros culturalmente competentes podem melhorar os resultados dos pacientes e reduzir a desigualdade na saúde.

**(2)** Os programas de graduação devem treinar enfermeiros para que se tornem enfermeiros culturalmente competentes.

**(3)** As habilidades linguísticas e o encontro cultural estão relacionados a uma maior competência cultural.

## Introdução

 Fatores culturais podem influenciar de forma crítica o relacionamento entre enfermeiro e paciente. Embora seja importante que os enfermeiros sejam clínica e tecnicamente competentes, a competência cultural (CC) permite que os profissionais de saúde se envolvam com pacientes culturalmente diversos de uma forma que respeite sua identidade cultural, permitindo assim uma prática segura e um atendimento de alta qualidade. Ela exige que os enfermeiros desenvolvam uma consciência de sua própria realidade cultural, bem como das atitudes e dos comportamentos que eles trazem para a assistência de enfermagem; desenvolvam um profundo respeito e aceitação das diferenças do paciente; e evitem que suas crenças pessoais exerçam uma influência indevida sobre aqueles cuja visão de mundo não é idêntica à sua ^(^ ^1^ ^)^ . 

 Cuidar de pacientes culturalmente diversos faz parte da rotina diária da maioria dos enfermeiros europeus. Espera-se que os profissionais de enfermagem ofereçam atendimento adequado a todos os pacientes, famílias e comunidades, garantindo que seus direitos sejam respeitados, independentemente de identificadores socioculturais como raça, etnia, gênero, idade e nacionalidade, entre outros ^(^ ^2^ ^-^ ^3^ ^)^ . A CC foi reconhecida como uma ponte que permite que os enfermeiros se conectem a outras culturas e compreendam as habilidades necessárias para oferecer um atendimento cultural seguro e centrado no paciente e na família ^(^ ^1^ ^)^ . 

 Para garantir a equidade na saúde para todos, os enfermeiros devem evitar atitudes etnocêntricas e preconceitos culturais, tornar-se sensíveis às necessidades de cada paciente, prestar atenção à comunicação verbal e não verbal, criar um relacionamento, envolver-se em negociações culturais e fazer esforços para promover o empoderamento do paciente, criando assim uma sensação de segurança no ambiente de saúde ^(^ ^4^ ^)^ . No entanto, cuidar de uma população de pacientes culturalmente diversa pode ser um desafio e requer preparação e treinamento adequados ^(^ ^5^ ^)^ . 

 É imperativo que a CC seja integrada ao ensino de enfermagem para ajudar os alunos a desenvolver uma prática culturalmente consciente e segura ^(^ ^6^ ^-^ ^7^ ^)^ . De fato, intervenções educacionais anteriores que buscavam integrar a CC no currículo de enfermagem foram eficazes para melhorar a CC em estudantes de enfermagem ^(^ ^8^ ^-^ ^9^ ^)^ . Em uma revisão sistemática ^(^ ^10^ ^)^ , os autores evidenciaram que o treinamento em CC teve um impacto positivo na CC dos profissionais de saúde e foi associado ao aumento da satisfação do paciente. Portanto, é importante projetar e implementar atividades educacionais criativas e baseadas em evidências que promovam os resultados do aprendizado de CC como parte dos programas de graduação em enfermagem ^(^ ^11^ ^-^ ^12^ ^)^ . 

 No contexto europeu, as instituições de ensino superior (IES) estão cada vez mais conscientes da necessidade de atender às diversas populações de pacientes. Especificamente, foi estabelecido que os currículos de enfermagem devem fornecer uma base para o desenvolvimento de CC que permita a aquisição de conhecimentos, habilidades e atitudes ^(^ ^13^ ^)^ . No entanto, a integração da CC no currículo dos cursos de graduação em enfermagem na Europa ainda é desigual ^(^ ^14^ ^-^ ^15^ ^)^ . Uma análise de escopo dos currículos de enfermagem na Espanha, Bélgica, Portugal e Turquia revelou uma abordagem fragmentada e não consistente do conceito. Por exemplo, na Espanha, apenas 4 dos 94 programas de graduação em enfermagem ministrados no ano acadêmico de 2018-2019 integraram a CC como parte de seus currículos ^(^ ^16^ ^)^ . Isso está de acordo com a opinião generalizada de que os currículos de enfermagem europeus carecem de conteúdo detalhado sobre CC ^(^ ^1^ ^)^ . No entanto, antes de implementar novas estratégias para abordar esse problema, é importante medir e analisar o nível atual de CC dos estudantes de enfermagem europeus. Portanto, o principal objetivo deste estudo foi avaliar o nível de CC de uma população de estudantes de graduação em enfermagem de quatro IES europeias. 

## Método

### Desenho do estudo e participantes

 Este estudo faz parte de um projeto maior financiado pela UE intitulado “Enfermagem Transcultural: Uma Prioridade Europeia, uma Responsabilidade Profissional para o Ensino Superior” (“ *Transcultural Nursing: A European Priority, a Professional Responsibility for Higher Education* ”, TC-NURSE), que se concentra na diversidade cultural, linguística e religiosa e promove a apropriação de valores compartilhados, a igualdade, a não discriminação e a inclusão social por meio da educação e do treinamento em nível de ensino superior. 

 Foi realizado um estudo transversal de CC em uma amostra de alunos do primeiro ao quarto ano de graduação em enfermagem de quatro universidades europeias ( *Universidad San Jorge* , Espanha; *Istanbul Aydin University* , Turquia; *Artis Plantin Hogeschool Antwerpen* , Bélgica; Instituto Politécnico de Portalegre, Portugal). Todas as universidades incluídas no estudo implementaram programas de internacionalização de alunos para promover a conscientização global, a compreensão intercultural e a colaboração internacional. Como resultado, todas elas estavam ligadas à mobilidade internacional de estudantes e se beneficiaram de várias iniciativas de internacionalização, inclusive o programa ERASMUS. 

Foi usada uma técnica de amostragem universal, ou seja, todos os estudantes de graduação em enfermagem registrados em uma das universidades foram convidados a participar do estudo. A amostra do estudo incluiu 168 estudantes de enfermagem de quatro programas de graduação em enfermagem.

Os critérios de inclusão para a seleção dos participantes foram os seguintes: a) estudantes de graduação em enfermagem registrados em uma das IES participantes; b) saber ler em inglês. Excluímos de nossa amostra os participantes que não conseguiam se comunicar no idioma oficial de cada país ou em inglês e aqueles que se recusaram a dar o consentimento informado.

 Um *e-mail* introdutório contendo o *link* para uma pesquisa eletrônica confidencial foi enviado a todos os estudantes de enfermagem por *e-mail* da universidade, explicando o objetivo do estudo, garantindo o anonimato e a confidencialidade das respostas e convidando-os a participar. Um lembrete foi enviado duas semanas depois. O convite e a coleta de dados foram realizados por alguns dos pesquisadores do estudo. 

### Coleta de dados

Os dados foram coletados entre outubro e novembro de 2021 nas quatro universidades participantes.

#### Variáveis sociodemográficas

O primeiro questionário, autoconstruído, continha 18 itens e foi elaborado para descrever as características sociodemográficas e o histórico cultural dos participantes. Coletamos informações sobre a idade, o sexo, a ocupação, a raça/etnia, a religião, o nível socioeconômico, o ano de estudo, a experiência de trabalho clínico, o treinamento anterior em CC e a experiência de trabalho com clientes de diversas origens culturais, entre outras características.

#### Sensibilidade Intercultural

 A Escala de Sensibilidade Intercultural ( *Intercultural Sensitivity Scale* , ISS) contém 24 itens ^(^ ^17^ ^)^ . Essa ferramenta foi projetada para avaliar a competência em comunicação intercultural e integra tanto a atitude intercultural quanto as habilidades comportamentais. Ela se baseia em uma escala Likert de 5 pontos (5 = concordo totalmente, 4 = concordo, 3 = incerto, 2 = discordo e 1 = discordo totalmente). Ele consiste em cinco fatores ou construtos nos quais as afirmações se baseiam: envolvimento na interação (7 itens), respeito pelas diferenças culturais (6 itens), confiança na interação (5 itens), prazer na interação (3 itens) e atenção na interação (3 itens). A pontuação geral da ISS varia entre 24 e 120, sendo que pontuações mais altas indicam maior competência intercultural. Essa escala demonstrou alta consistência interna com coeficiente de confiabilidade de 0,86. 

#### Competência Cultural

 A Ferramenta de Avaliação de Competência Cultural ( *Cultural Competence Assessment Tool* , CCATool) em sua versão para estudantes foi usado para medir a CC em nossa amostra ^(^ ^18^ ^)^ . Esse instrumento é baseado no modelo de desenvolvimento de competência cultural ^(^ ^19^ ^)^ . Ele inclui quatro seções que medem a consciência cultural, o conhecimento cultural, a sensibilidade cultural e a prática cultural, além de uma seção final de dados sociodemográficos. Essa seção final foi removida da pesquisa eletrônica, pois medimos as características sociodemográficas e culturais dos nossos participantes por meio de um questionário autoconstruído. Cada uma das quatro seções contém 10 declarações medidas em uma escala Likert de 4 pontos com a qual os respondentes devem concordar ou discordar (1= discordo totalmente, 2= discordo, 3= concordo, 4= concordo totalmente). Além disso, escalas visuais analógicas (EVAs) foram incluídas para permitir que os participantes autoavaliassem sua consciência cultural, conhecimento, sensibilidade e prática. A validade foi confirmada por meio de painéis de especialistas; o coeficiente de confiabilidade alfa de Cronbach foi de 0,70 ou mais ^(^ ^20^ ^-^ ^22^ ^)^ . 

#### Consciência Cultural

 Desenvolvida em 2003 e revisada em 2014 ^(^ ^23^ ^-^ ^24^ ^)^ , a Escala de Consciência Cultural ( *Cultural Awareness Scale* , CAS) é uma escala de 36 itens, medida em uma escala Likert de 7 pontos, e inclui cinco construtos ou categorias de consciência cultural, a saber, experiência educacional geral, consciência cognitiva, questões de pesquisa, comportamento/conforto com interações e atendimento ao paciente/questões clínicas. A confiabilidade da consistência interna da CAS foi demonstrada anteriormente em estudantes de enfermagem ^(^ ^9^ ^,^ ^23^ ^)^ . 

### Análise de dados

 Foram calculadas estatísticas descritivas (média, desvio padrão e porcentagem) para descrever as características da amostra. A homogeneidade e a distribuição dos resultados foram examinadas. Quando uma escala apresentava distribuição normal, o teste t de Student para amostras independentes ou a ANOVA eram usados nas comparações; testes não paramétricos (teste U de Mann-Whitney ou Kruskal-Wallis) eram usados quando uma escala não apresentava distribuição normal. Todas as análises estatísticas foram realizadas com o *software* Pacote Estatístico para Ciências Sociais ( *Statistical Package for the Social Sciences)* (versão 21.0, SPSS Institute Inc., Chicago, IL, EUA). O nível de significância foi definido como α = 0,05. 

### Considerações éticas

 Os participantes elegíveis foram informados sobre os objetivos e a finalidade do projeto, e a participação no estudo foi voluntária. O consentimento informado para participar do estudo foi presumido quando os participantes preencheram e enviaram a pesquisa eletrônica. A permissão para usar as ferramentas de medição aplicadas neste estudo foi solicitada e concedida pelos autores. O presente estudo foi aprovado pelo Comitê de Ética em Pesquisa da *Universidad de San Jorge* (16-2019) e foi realizado de acordo com os princípios da Declaração de Helsinque. 

## Resultados

### Características dos participantes

 As características sociodemográficas e culturais dos participantes são mostradas na Tabela 1 . Um total de 168 estudantes de graduação em enfermagem dos locais de estudo responderam à pesquisa eletrônica. Especificamente, 63 estudantes participaram da Espanha (37,5%), 56 da Turquia (33,5%), 32 da Bélgica (19%) e 17 de Portugal (10%). A maioria dos participantes era do sexo feminino (82%), solteira (86,3%) e a idade média era de 21,6±3,93 anos. Dos nossos participantes, 41% estavam no primeiro ou segundo ano de estudo, enquanto 59% estavam no terceiro ou último ano. Com relação às suas características culturais, a grande maioria dos nossos alunos se descreveu como sendo de origem branca (92,3%); apenas 3,6% dos nossos alunos se descreveram como sendo negros, 3% como sendo asiáticos e 1,2% como sendo árabes. Em termos de status socioeconômico, nossos resultados mostram que a maioria dos participantes era de classe média (85,1%) e residia em uma área urbana (84,5%). Metade dos nossos participantes não tinha experiência clínica anterior e a maioria deles nunca havia participado de um curso de treinamento em CC (80,45%). Além disso, apenas 14,3% dos estudantes de enfermagem haviam morado no exterior por pelo menos três meses. É interessante notar que aproximadamente 60% dos alunos se descreveram como pertencentes a uma família ou a um grupo de amigos culturalmente diversificado e metade da nossa amostra afirmou ter cuidado de pacientes de diversas origens culturais durante seus estágios (51,8%). 

**Tabela 1:** - Características sociodemográficas e culturais (n = 168). Zaragoza, Espanha; Portalegre, Portugal; Istambul, Turquia; Antuérpia, Bélgica, 2021

**Variável**	**Número**	**Porcentagem (%)**
Idade. Média±DP ^*^	21,63±3,93	
<21 anos	67	39,9
≥21 anos	101	60,1
Gênero		
Feminino	139	82,7
Masculino	29	17,3
País do estudo		
Espanha	63	37,5
Turquia	56	33,3
Bélgica	32	19,0
Portugal	17	10,1
Ano do estudo		
Primeiro ano	55	32,7
Segundo ano	14	8,3
Terceiro ano	62	36,9
Quarto ano	37	22,0
Estado civil		
Solteiro	145	86,3
Casado/Parceiro	23	13,7
Ocupação		
Apenas estuda	131	78,0
Trabalha e estuda	37	22,0
Raça/Etnia		
Raça Árabe	2	1,2
Caucasiano/Branco	155	92,3
Negro	6	3,6
Asiático	5	3,0
Afiliação religiosa		
Islamismo	62	36,9
Catolicismo	62	36,9
Outro	19	11,3
Ateísmo	25	14,9
Ambiente residencial		
Urbano	142	84,5
Rural	26	15,5
Status social		
Classe social mais baixa	4	2,4
Classe social média	143	85,1
Classe social alta	21	12,5
Experiência em trabalho clínico		
Não	84	50,0
Sim	84	50,0
Pertencer a uma família ou a um grupo de amigos culturalmente diverso		
Não	63	37,5
Sim	105	62,5
Treinamento prévio em competência cultural		
Não	135	80,4
Sim	33	19,6
Trabalho voluntário anterior/atual com pacientes de diversas origens culturais e organizações		
Não	107	63,7
Sim	61	36,3
Experiência no atendimento a pacientes de diversas origens culturais		
Não	81	48,2
Sim	87	51,8
Morou/estudou no exterior por pelo menos 3 meses		
Não	144	85,7
Sim	24	14,3

^*^ DP *=* Desvio-Padrão

### Sensibilidade Intercultural

 Os resultados da ISS são apresentados na Tabela 2 . A pontuação média geral foi de 95,80±10,86. Levando em conta o número de itens nas subescalas da ISS, as pontuações médias foram um pouco mais altas para as subescalas envolvimento na interação (27,85±3,51), respeito pelas diferenças culturais (25,27±4,08) e prazer na interação (12,47±2,69); comparativamente, foram observadas pontuações médias mais baixas nas subescalas confiança na interação (18,75±2,92) e atenção na interação (11,61±1,88). 

Não foram encontradas diferenças significativas nas pontuações das subescalas confiança na interação, prazer na interação e atenção à interação entre os alunos por país de estudo. Os alunos portugueses apresentaram pontuações mais altas nas subescalas envolvimento na interação (29,70±2,84; p=0,029) e respeito pelas diferenças culturais (p=0,000); os alunos belgas também obtiveram pontuações mais altas na subescala respeito pelas diferenças culturais em comparação com os alunos espanhóis e turcos.

**Tabela 2:** - Resultados da Escala de Sensibilidade Intercultural classificados por país de estudo (n = 168). Zaragoza, Espanha; Portalegre, Portugal; Istambul, Turquia; Antuérpia, Bélgica, 2021

**Variável**	**Geral**	**Espanha**	**Turquia**	**Bélgica**	**Portugal**	**p-valor**
	**Média±DP** ^*^	**Média±DP** ^*^	**Média±DP** ^*^	**Média±DP** ^*^	**Média±DP** ^*^	
Geral (24 itens)	95,80±10,86	96,49±10,89	94,21±10,62	94,40 ±10,83	101,11±10,64	0,109 ^†^
Engajamento de interação (7 itens)	27,85±3,51	27,77±3,92	28,08±3,28	26,62±3,02	29,70±2,84	**0,029** ^†^
Respeito pelas diferenças culturais (6 itens)	25,27±4,08	25,12±3,89	24,14±4,38	26,18±3,66	27,88±3,07	**0,000** ^‡^
Confiança na interação (5 itens)	18,57±2,92	19,14±2,72	18,05±2,75	18,37±2,99	18,58±3,87	0,274 ^‡^
Prazer com a interação (3 itens)	12,47±2,69	12,74±1,93	12,01±3,30	12,46±2,69	13,00±2,91	0,578 ^‡^
Atenção à interação (3 itens)	11,61±1,88	11,69±1,90	11,91±1,80	10,75±1,91	11,94±1,63	0,068 ^‡^

^*^ DP = Desvio-Padrão;

^†^ ANOVA;

^‡^ Kruskal-Wallis

### Competência Cultural

 Os resultados da CCATool foram apresentados na Tabela 3 . Os participantes obtiveram pontuação mais alta nas dimensões consciência cultural (4,06) e prática cultural (4,01), e um pouco mais baixa em competência cultural (3,81), conhecimento cultural (3,69) e sensibilidade cultural (3,46). 

As pontuações obtidas pelos alunos das diferentes universidades europeias foram semelhantes. Não foram encontradas diferenças significativas entre os estudantes de enfermagem por país de estudo, com exceção da primeira dimensão, consciência cultural. Os resultados indicam que existe uma diferença estatisticamente significativa nas pontuações obtidas pelos alunos em consciência cultural (Espanha-Turquia; p=0,023).

**Tabela 3:** - Resultados da Ferramenta de Avaliação de Competência Cultural classificados por país dos alunos (n = 168). Zaragoza, Espanha; Portalegre, Portugal; Istambul, Turquia; Antuérpia, Bélgica, 2021

**Variável**	**Geral**	**Espanh**	**Turquia**	**Bélgica**	**Portugal**	**p valor**
	**Média±DP** ^*^	**Média±DP** ^*^	**Média±DP** ^*^	**Média±DP** ^*^	**Média±DP** ^*^	
Competência cultural (40 itens)	3,81 (0,34)	3,79 (0,29)	3,84 (0,43)	3,77 (0,29)	3,82 (0,24)	0,768 ^†^
Consciência cultural (10 itens)	4,06 (0,47)	4,01 (0,33)	4,14 (0,64)	3,99 (0,42)	4,12 (0,28)	**0,023** ^‡^
Conhecimento cultural (10 itens)	3,69 (0,44)	3,64 (0,40)	3,69 (0,53)	3,74 (0,39)	3,77 (0,37)	0,687 ^†^
Sensibilidade cultural (10 itens)	3,46 (0,43)	3,47 (0,35)	3,53 (0,53)	3,38 (0,39)	3,37 (0,40)	0,362 ^†^
Prática cultural (10 itens)	4,01 (0,49)	4,05 (0,53)	4,00 (0,52)	3,96 (0,41)	4,01 (0,39)	0,798 ^‡^

^*^ DP = Desvio-Padrão;

^†^ ANOVA;

^‡^ Kruskal-Wallis

### Conscientização Cultural

 A Tabela 4 mostra os resultados da CAS, em geral e por categorias, por país de estudo. A pontuação média para essa escala foi 188,07±21,45. Os estudantes portugueses apresentaram as pontuações mais altas em geral e em todas as categorias, com uma diferença estatisticamente significativa entre eles e o restante dos estudantes de enfermagem na pontuação do CAS (p=0,006). Os estudantes belgas obtiveram a pontuação mais baixa na experiência de educação geral, embora não tenham sido encontradas diferenças significativas nessa categoria. Houve uma diferença estatisticamente significativa entre os estudantes de enfermagem espanhóis e turcos em consciência cognitiva (p=0,005), e entre os estudantes espanhóis e belgas em questões de pesquisa (p=0,004). Não foi observada nenhuma diferença em comportamentos/conforto entre os quatro grupos de estudantes de enfermagem. Por fim, foi observada uma diferença estatisticamente significativa na categoria atendimento ao paciente/questões clínicas (Portugal 31,0±2,03; Espanha 28,26±3,15; p=0,009). 

**Tabela 4:** - Resultados da Escala de Consciência Cultural por país de estudo (n = 168). Zaragoza, Espanha; Portalegre, Portugal; Istambul, Turquia; Antuérpia, Bélgica, 2021

**Variável**	**Geral**	**Espanha**	**Turquia**	**Bélgica**	**Portugal**	**p valor**
	**Média±DP** ^*^	**Média±DP** ^*^	**Média±DP** ^*^	**Média±DP** ^*^	**Média±DP** ^*^	
Experiência em educação geral (14 itens)	71,58±11,60	72,04±11,54	70,26±10,57	69,78±10,87	77,58±14,97	0,104 ^†^
Consciência cognitiva (7 itens)	36,25±6,45	37,66±4,32	33,75±7,45	36,81±7,50	38,17±5,24	**0,005** ^‡^
Questões de pesquisa (4 itens)	20,63±3,03	21,65±1,89	20,14±3,40	19,15±3,65	21,23±2,61	**0,004** ^‡^
Comportamento/ Conforto com interações (6 itens)	31,31±6,39	30,88±6,11	30,91±7,01	31,71±6,40	33,47±5,20	0,467 ^†^
Cuidados com o paciente/questões clínicas (5 itens)	28,29±4,68	28,26±3,15	27,91±5,79	27,59±5,65	31,00±2,03	**0,009** ^‡^
Geral (36 itens)	188,07±21,45	190,52±14,96	182,98±25,02	185,06±24,72	201,47±16,66	**0,006** ^‡^

^*^ DP = Desvio-Padrão;

^†^ ANOVA;

^‡^ Kruskal-Wallis

Foi realizada uma análise bivariada da relação entre o nível de sensibilidade intercultural, CC e consciência cultural dos alunos e seu ano de estudo. Os alunos foram divididos em dois grupos: 1) alunos do primeiro e segundo anos (41%) e 2) alunos do terceiro e quarto anos (59%). Não foram encontradas diferenças significativas entre os dois grupos para o CAS (p=0,133), ISS (p=0,570) e CCATool (p=0,624) (dados não apresentados nas tabelas).

## Discussão

 As características sociodemográficas de nossos participantes refletiram as da maioria dos estudantes de graduação em enfermagem registrados nas quatro universidades participantes. Com relação às características culturais dos alunos, achamos interessante o fato de que a maioria se descreveu como pertencente a uma família ou grupo de amigos culturalmente diverso. Isso provavelmente é um reflexo da sociedade nos quatro países deste estudo, mas também pode ser devido ao fato de os alunos adotarem uma compreensão mais ampla da diferença cultural, que envolve não apenas raça e etnia, mas também outras características, como idade, religião, orientação sexual e área geográfica ^(^ ^24^ ^)^ . 

 Como um todo, nossos alunos alcançaram um alto nível de sensibilidade intercultural. Resultados semelhantes foram obtidos por outros autores em três amostras de estudantes de profissões de saúde na Turquia ^(^ ^25^ ^-^ ^26^ ^)^ e na Austrália ^(^ ^27^ ^)^ . Especificamente, observou-se que tanto os estudantes de medicina quanto os de enfermagem que interagiram com pessoas de culturas diferentes e falavam um segundo idioma apresentaram níveis mais altos de sensibilidade intercultural ^(^ ^25^ ^)^ . É interessante notar que todos os alunos de nossa amostra falavam pelo menos mais um idioma (inglês), e mais da metade deles pertencia a um grupo culturalmente diverso de amigos e/ou familiares e tinha experiência em cuidar de pacientes de culturas minoritárias em seus estágios clínicos. 

 O nível de competência cultural dos nossos participantes, medido pela CCATool, foi moderado. Em um estudo com 295 estudantes de graduação na Finlândia ^(^ ^21^ ^)^ obtivemos resultados semelhantes, com a diferença de que os nossos alunos obtiveram níveis mais altos de conhecimento cultural, enquanto os deles apresentaram níveis mais altos de competência cultural. Foi interessante observar que, embora mais de 80% desses participantes tenham tido algum tipo de treinamento formal em CC, apenas 20% da nossa amostra já havia participado de um curso de CC. Entretanto, em ambos os casos foi estabelecida uma associação entre a frequência de encontros culturais e habilidades linguísticas e níveis mais altos de competência cultural. 

 Nossos alunos alcançaram um nível moderado de consciência cultural. Em um estudo transversal recente ^(^ ^27^ ^)^ envolvendo uma amostra de enfermeiros e estudantes de enfermagem, foram observados altos níveis de consciência cultural entre a amostra (média = 4,42; DP = 0,45). Isso corroboraria nossa observação inicial de que a CC não está integrada nos currículos ou, pelo menos, não é explicitada aos alunos quando as questões culturais são abordadas em sala de aula. 

 Analisamos as pontuações dos alunos nas três escalas separadamente por país de estudo. Em geral, os estudantes portugueses e belgas obtiveram pontuações mais altas do que os espanhóis e turcos em todas as escalas. É possível que esses resultados realmente reflitam uma diferença entre os quatro grupos de estudantes de enfermagem. No entanto, o fato de um número menor de estudantes de enfermagem da Bélgica e de Portugal ter participado deste estudo pode ter resultado em uma amostra de estudantes com um interesse ou inclinação especial para a CC e, portanto, potencialmente apresentando um nível mais alto de consciência e competência de sensibilidade cultural. O fato de os estudantes de profissões da área de saúde em geral, e de enfermagem em particular, atingirem altos níveis de sensibilidade intercultural não é surpreendente. Os itens contidos no ISS fazem referência a atitudes e/ou qualidades esperadas de um enfermeiro, como “Tenho a mente aberta para pessoas de culturas diferentes” e “Respeito a maneira como as pessoas de culturas diferentes se comportam”. É possível, é claro, que a pontuação média obtida por nossos estudantes de enfermagem no ISS seja um reflexo verdadeiro de seu nível de sensibilidade intercultural. Entretanto, também é possível que suas respostas sejam parcialmente influenciadas pela imagem projetada de como uma enfermeira qualificada deve ser ou se comportar. Afinal de contas, qualidades como respeito, mente aberta e tolerância são frequentemente associadas à profissão de enfermeiro, e todas elas seriam necessárias para demonstrar um alto nível de sensibilidade cultural ^(^ ^28^ ^)^ . 

Independentemente da motivação por trás das respostas dos alunos, essa é uma descoberta muito importante e que deve ser considerada na elaboração de materiais e atividades de ensino e aprendizagem relacionadas à CC. Com base em nossas descobertas, pode-se argumentar que os alunos de graduação em enfermagem são um terreno fértil para continuar a desenvolver o conhecimento, as habilidades e as atitudes necessárias para se tornar um profissional culturalmente competente. Avaliar o nível de competência inicial dos alunos é fundamental para a elaboração de resultados de aprendizagem que sejam alcançáveis, mas desafiadores para um grupo de alunos; para a preparação de materiais e atividades de ensino e aprendizagem que permitam que os alunos adquiram novos conhecimentos, habilidades e atitudes e continuem a desenvolver os existentes; e para a avaliação justa e adequada da aprendizagem dos alunos. Portanto, recomendamos que os professores universitários e tutores clínicos levem em conta o nível inicial de sensibilidade intercultural dos alunos ao planejar suas sessões de ensino.

 Fatores como a competência linguística e o encontro cultural são frequentemente associados a níveis mais altos de competência cultural ^(^ ^29^ ^-^ ^30^ ^)^ . Todos os nossos alunos sabiam falar inglês e pelo menos metade deles conseguiu interagir com pessoas de diversas origens culturais durante os estágios clínicos ou como parte de um grupo diversificado de amigos ou familiares. Essas características podem ter resultado em uma superestimação da sensibilidade intercultural, da CC e da consciência cultural de nossos participantes. Dito isso, parece claro que promover as habilidades linguísticas de nossos alunos e permitir que eles vivenciem encontros culturais, tanto como parte de seus programas de estudo quanto como atividades complementares, como escolas de verão e cursos eletivos com foco na internacionalização, pode ser útil para aumentar seu nível de CC. Portanto, argumentamos que devem ser oferecidas oportunidades para que os alunos de enfermagem interajam com pessoas de diversas origens culturais, desenvolvam habilidades linguísticas e ofereçam possibilidades de internacionalização ^(^ ^31^ ^)^ . 

 Foi muito interessante observar que não houve diferenças significativas nos resultados da ISS, da CCATool e da CAS em relação ao ano de estudo dos alunos. Esperávamos que nossos alunos do terceiro e último ano atingissem um nível mais alto de CC do que os do primeiro e segundo anos. Isso se baseou na suposição de que os alunos do terceiro e quarto anos estão expostos a uma gama maior e mais ampla de oportunidades de aprendizado, incluindo programas de mobilidade internacional, internacionalização em casa, estágios clínicos e outros. Essa constatação corrobora nossa opinião de que a CC não está suficientemente integrada aos programas europeus de graduação em enfermagem; isso também sugere que as atuais oportunidades de aprendizado obrigatórias e eletivas não são eficazes ou não são aproveitadas pelos alunos. Estudos anteriores demonstraram que a integração de módulos de CC e cursos de treinamento no currículo é eficaz para promover a CC entre os estudantes de graduação em enfermagem ^(^ ^32^ ^-^ ^33^ ^)^ . Sugerimos que essas atividades sejam elaboradas cuidadosamente, com base no nível inicial de CC dos alunos, e incluam elementos que tenham sido fortemente associados a uma CC mais alta, incluindo habilidades linguísticas, encontros culturais e oportunidades de internacionalização. 

Houve uma diferença estatisticamente significativa entre os estudantes de enfermagem espanhóis e turcos com relação à conscientização cognitiva. Há vários fatores que podem contribuir para a variação nos níveis de consciência cognitiva da competência cultural entre os estudantes de enfermagem, incluindo o histórico e as experiências pessoais dos estudantes, a preparação educacional, as colocações clínicas, a eficácia dos métodos de ensino e dos materiais instrucionais usados para transmitir conceitos de CC, as atitudes, as crenças e as práticas de ensino do corpo docente de enfermagem, a habilidade e a capacidade de autorreflexão e pensamento crítico dos estudantes e, por último, mas não menos importante, a exposição ao treinamento em CC.

De modo geral, a interação de fatores pessoais, experiências educacionais, influência do corpo docente, exposição clínica e aprendizado autodirigido contribui para a variação nos níveis de consciência cognitiva da competência cultural entre os estudantes de enfermagem. Esforços para aprimorar a educação sobre competência cultural e criar ambientes de aprendizagem favoráveis podem ajudar a eliminar essas disparidades e garantir que todos os alunos desenvolvam a conscientização e as habilidades necessárias para prestar um atendimento culturalmente sensível.

Os principais resultados deste estudo mostraram que os estudantes de enfermagem de diferentes universidades europeias têm um alto grau de sensibilidade intercultural, o que sugere que os programas de treinamento das escolas de enfermagem estão tentando promover algumas áreas de competência cultural, como entender, apreciar e respeitar pessoas de outras culturas. No entanto, a integração bem-sucedida da CC nos currículos de enfermagem requer uma abordagem abrangente que pode incluir várias estratégias e considerações, incluindo: 1) Definir claramente os objetivos de aprendizado específicos relacionados à competência cultural dentro do currículo de enfermagem; 2) Infundir o conteúdo de CC em todo o currículo de enfermagem, em vez de confiná-lo a um único curso ou módulo; 3) Oferecer oportunidades para que os alunos se envolvam em experiências do mundo real que os exponham a diversas culturas e comunidades, por exemplo, como parte de colocações clínicas em ambientes culturalmente diversos; 4) Oferecer experiências de imersão cultural, como rodízios clínicos internacionais ou projetos baseados na comunidade em bairros multiculturais; 5) Colaborar com outras disciplinas da área de saúde para incorporar a educação interprofissional com foco na competência cultural; 6) Fornecer ao corpo docente treinamento e recursos para ensinar com eficácia os conceitos de competência cultural e facilitar discussões significativas sobre diversidade cultural em sala de aula; 7) Integrar ferramentas e métodos de avaliação para avaliar a CC dos alunos durante todo o programa de enfermagem; 8) Estabelecer parcerias com organizações comunitárias, centros culturais e agências de saúde que atendam a populações diversas e colaborar com as partes interessadas que possam oferecer oportunidades valiosas para que os alunos interajam e aprendam com indivíduos de diferentes origens culturais; 9) Oferecer aos alunos acesso a recursos e serviços de apoio, como oficinas de competência cultural, módulos de treinamento on-line e materiais de treinamento de sensibilidade cultural; e 10) Avaliar regularmente a eficácia das iniciativas de CC no currículo de enfermagem e solicitar feedback dos alunos, do corpo docente e dos parceiros da comunidade.

De modo geral, esta pesquisa pode contribuir para o avanço do conhecimento científico ao aprimorar nossa compreensão do desenvolvimento da competência cultural, das intervenções educacionais, das variações interculturais, das barreiras e facilitadores e dos métodos de avaliação na educação e na prática da enfermagem.

 Há algumas limitações neste estudo que devem ser observadas. Em primeiro lugar, o tamanho da pesquisa eletrônica foi uma preocupação (163 itens); alguns alunos podem ter achado o preenchimento oneroso, resultando em algum grau de viés de resposta. No entanto, estávamos interessados em medir a sensibilidade intercultural dos alunos, bem como seu nível percebido de competência cultural; a ferramenta CAS nos permitiu avaliar também a percepção ou a conscientização dos alunos sobre a educação e o treinamento em competência cultural recebidos em suas respectivas universidades. Em segundo lugar, considerando o universo total de estudantes de enfermagem registrados nos locais de estudo, o tamanho da nossa amostra foi limitado. Isso é particularmente verdadeiro no caso da *Artis Plantin Hogeschool Antwerpen* e do Instituto Politécnico de Portalegre e pode explicar, até certo ponto, as pontuações mais altas dos alunos belgas e portugueses em alguns dos questionários. Isso poderia ter sido evitado com a adaptação e a tradução das ferramentas para os quatro idiomas do estudo, mas essa opção foi descartada devido a restrições de tempo e orçamento. Por fim, o fato de todos os nossos alunos falarem inglês, e o fato de falar um segundo idioma ter sido associado a níveis mais altos de CC, dificulta a generalização dos nossos resultados para toda a população de estudantes de graduação em enfermagem nos quatro países do estudo. 

## Conclusão

Os estudantes de graduação em enfermagem de quatro universidades europeias alcançaram um alto nível de sensibilidade intercultural e um nível moderado de CC e consciência cultural. Portanto, é altamente necessário que os educadores de enfermagem levem em consideração o nível de CC dos alunos antes de projetar e implementar qualquer programa de estudo relacionado.

Os estudantes de enfermagem não são uma tela em branco, mas um terreno fértil no qual podem continuar a desenvolver o conhecimento, as habilidades e as atitudes necessárias para se tornarem profissionais com competência cultural. Dessa forma, os futuros programas e atividades de ensino e aprendizagem relacionados à CC devem ser cuidadosamente elaborados e incluir elementos que tenham sido associados à CC aprimorada, inclusive habilidades linguísticas, encontros culturais e oportunidades de internacionalização.
